# Linking Brain Age Gap to Mental and Physical Health in the Berlin Aging Study II

**DOI:** 10.3389/fnagi.2022.791222

**Published:** 2022-07-22

**Authors:** Philippe Jawinski, Sebastian Markett, Johanna Drewelies, Sandra Düzel, Ilja Demuth, Elisabeth Steinhagen-Thiessen, Gert G. Wagner, Denis Gerstorf, Ulman Lindenberger, Christian Gaser, Simone Kühn

**Affiliations:** ^1^Department of Psychology, Humboldt-Universität zu Berlin, Berlin, Germany; ^2^Lise Meitner Group for Environmental Neuroscience, Max Planck Institute for Human Development, Berlin, Germany; ^3^Center for Lifespan Psychology, Max Planck Institute for Human Development, Berlin, Germany; ^4^Division of Lipid Metabolism, Department of Endocrinology and Metabolic Diseases, Corporate Member of Freie Universität Berlin and Humboldt-Universität zu Berlin, Charité – Universitätsmedizin Berlin, Berlin, Germany; ^5^Berlin Institute of Health at Charité – Universitätsmedizin Berlin, BCRT-Berlin Institute of Health Center for Regenerative Therapies, Berlin, Germany; ^6^German Socio-Economic Panel Study (SOEP), Berlin, Germany; ^7^Federal Institute for Population Research (BiB), Berlin, Germany; ^8^Structural Brain Mapping Group, Department of Psychiatry and Neurology, Jena University Hospital, Jena, Germany; ^9^Department of Psychiatry and Psychotherapy, University Clinic Hamburg Eppendorf, Hamburg, Germany

**Keywords:** aging, brain age gap, cognition, mental health, Berlin Aging Study II (BASE-II)

## Abstract

From a biological perspective, humans differ in the speed they age, and this may manifest in both mental and physical health disparities. The discrepancy between an individual’s biological and chronological age of the brain (“brain age gap”) can be assessed by applying machine learning techniques to Magnetic Resonance Imaging (MRI) data. Here, we examined the links between brain age gap and a broad range of cognitive, affective, socioeconomic, lifestyle, and physical health variables in up to 335 adults of the Berlin Aging Study II. Brain age gap was assessed using a validated prediction model that we previously trained on MRI scans of 32,634 UK Biobank individuals. Our statistical analyses revealed overall stronger evidence for a link between higher brain age gap and less favorable health characteristics than expected under the null hypothesis of no effect, with 80% of the tested associations showing hypothesis-consistent effect directions and 23% reaching nominal significance. The most compelling support was observed for a cluster covering both cognitive performance variables (episodic memory, working memory, fluid intelligence, digit symbol substitution test) and socioeconomic variables (years of education and household income). Furthermore, we observed higher brain age gap to be associated with heavy episodic drinking, higher blood pressure, and higher blood glucose. In sum, our results point toward multifaceted links between brain age gap and human health. Understanding differences in biological brain aging may therefore have broad implications for future informed interventions to preserve mental and physical health in old age.

## Introduction

The world’s population is growing older rapidly. It is expected that by 2050, every sixth living person will be aged 65 + and every 20th person aged 80 + (United Nations, 2019). To seize the opportunities that come with increasing longevity and the extension of individual life spans, it is imperative to foster successful aging, i.e., to maximize healthy and functional years among older adults (Urtamo et al., 2019). Aging itself is considered a risk factor for several prevalent conditions (Niccoli and Partridge, 2012). The incidence for Alzheimer’s disease, for instance, doubles every 5 years after age 65, ultimately affecting more than one out of three people above age 85 (Querfurth and LaFerla, 2010). The impeding burden by neurodegeneration and dementia brings brain health and successful cognitive aging into the main focus.

Aging is often accompanied by cognitive decline, particularly in the domains of episodic memory and executive functioning (Tucker-Drob et al., 2019). At the same time, cognitive aging is highly heterogeneous. Individual differences in developmental trajectories of cognitive aging are of considerable size, and an early age-related cognitive decline in some individuals is juxtaposed by many older adults who are able to maintain a high level of cognitive functioning into very old age (Nyberg et al., 2012; Lindenberger, 2014). A similar variation can be observed regarding the trajectories of physical health, which underscores that it is not chronological age *per se* that constitutes the major risk factor for morbidity, disability, and frailty, but a decline in tissue and organ functioning that results from a build-up of damage and limitations in somatic maintenance that occur throughout adulthood (Kirkwood, 2005; Ferrucci et al., 2018). This decline can be described as biological aging (Hamczyk et al., 2020). According to the biological aging perspective, age-related loss of functionality and resulting frailty begin once compensatory mechanisms are exhausted and the organism forfeits its resilience (Ferrucci and Fabbri, 2018).

Processes of biological aging themselves, however, are gradual and often begin long before apparent clinical manifestations. Finding a sensitive measure for biological aging is therefore an important step not only toward a better understanding of aging processes, but also toward identifying individuals at high risk for accelerated biological aging, who would benefit the most from early interventions so as to slow its progress, to counteract or buffer resulting functional losses, and to prolong health over the lifespan (Ferrucci et al., 2020). Current attempts focus predominantly on disparities between chronological age and biological age in order to identify individuals who age particularly fast and who would benefit from such interventions the most (Li et al., 2020). This is commonly achieved through normative models of biological aging that build on predictive analytics to predict chronological age from various biological data sources (Jia et al., 2017).

An easily applicable, effective, and robust method for such an endeavor has been proposed and validated over the recent years (Franke and Gaser, 2019): the brain age gap estimation method (brainAGE). BrainAGE utilizes a common voxel-based morphometry pipeline to train a classifier that learns to predict chronological age from structural magnetic resonance images (MRI). The classifier represents a normative model of brain aging (Dosenbach et al., 2010; Cole and Franke, 2017), and once trained, the classifier can be applied to new data so as to predict the chronological age of an individual from his or her brain characteristics. This prediction can reveal a discrepancy between an individual’s chronological age and his or her brain age, as derived from the normative training data. The estimated brain age gap has been interpreted as accelerated or decelerated brain aging relative to one’s chronological age. MRI-based brain age gap has been shown to be highly reliable, applicable in both human subjects and animal models, as well as sensitive to health variables and risk factors, and can be derived for adults across the entire lifespan (Franke and Gaser, 2012; Franke et al., 2013, 2014, 2016, 2018).

Over the past decade, a plethora of investigations have linked individual differences in brain age gap to physical and mental health traits as well as sociodemographic characteristics (Franke and Gaser, 2019). For instance, a higher discrepancy between brain age and chronological age has been found to be associated with drinking and smoking behavior, higher systolic and diastolic blood pressure, diabetes, depressive symptoms, and lower educational attainment (Franke et al., 2013, 2014; Steffener et al., 2016; Smith et al., 2019). Brain age gap has also been observed to predict the conversion from mild cognitive impairment to Alzheimer’s disease, and seems to be malleable to interventions (Gaser et al., 2013; Luders et al., 2016; Rogenmoser et al., 2018). Higher brain age gap is commonly observed in neurological and psychiatric disorders such as schizophrenia, multiple sclerosis, mild cognitive impairment, and dementia, and has a genetic foundation that overlaps with the genetic architecture of psychiatric phenotypes (Nenadić et al., 2017; Hajek et al., 2019; Kaufmann et al., 2019). One major aim of the present study is to establish brain-predicted age estimates in a cohort that has not yet been used in brain age research, and to replicate a set of previously reported mental and physical health associations. The great merit of replication is to potentially corroborate previous findings and foster their credibility through an inevitably differing approach. Our replication attempt can be considered a generative contribution to the field, as it will add information concerning the generalizability and validity of reported brain age associations.

It is an impeding question whether individuals with higher brain age compared to their chronological age also show signs of earlier cognitive decline. Several studies have reported correlations between brain age gap and cognitive variables (for a recent overview see Boyle et al., 2021), but interpretability is still compromised by methodological issues such as small sample sizes with reduced statistical power, confounding between brain age gap and chronological age (Smith et al., 2019), possible alpha error inflation through multiple null hypothesis significance testing, unclear replicability, and complex composite measures. Associations between brain age gap and cognitive measures in healthy participants have been reported for general cognitive status, verbal fluency, processing speed, and selective attention (Franke et al., 2013; Richard et al., 2018; Smith et al., 2019; Boyle et al., 2021), but effect sizes and also the number of tested cognitive variables suggest that associations are often subtle and ability-specific. Further work on the relationship between brain age gap and cognition is clearly needed.

Next to cognitive variables, it is currently also unclear how brain age gap relates to motivational choices and goal priorities, and to the future time perspective people hold for themselves. People who lead active and enriched lives and routinely engage in physically and intellectually demanding activities often show higher levels of cognitive functioning throughout older age, show less signs of brain atrophy, and are also less likely to develop dementia (Najar et al., 2019; Casaletto et al., 2020). Making plans for future activities has been shown to be dependent on an individual’s global perception of their overall time left, i.e., whether one’s own future is regarded as open-ended with many opportunities or rather close-ended and limited (Lang and Carstensen, 2002). An open-ended perspective has been linked to wellbeing and lower scores of depression (Kooij et al., 2018). On average, older adults perceive their future time as more limited (Lang and Carstensen, 2002), and as the amount of time left in life gets increasingly salient, motivational priorities are shifted so that emotional goals are favored over knowledge-related goals (Carstensen et al., 1999; Carstensen, 2006). As individuals grow older, they may also tend to emphasize short-term over long-term consequences of their current behavior (Orbell et al., 2004). Interestingly, subjective age, referring to the degree to which individuals experience themselves younger or older than their actual chronological age, has been linked to cognitive function in later life (Stephan et al., 2014), as well as to gray matter volume and brain aging (Kwak et al., 2018). Similarly, future time perspective and facets of the subjective health horizon have both been linked to episodic memory, metabolic health (Düzel et al., 2016) as well as to differences in specific gray matter regions of the brain among older adults (Düzel et al., 2018b). Associations with brain age gap, however, have not been tested before.

The present study investigates brain age gap in a subset of participants from the Berlin Aging Study II (BASE-II, *N* = 355), a developmental cohort study comprising 2,200 adult volunteers from the greater metropolitan area of Berlin, Germany (Bertram et al., 2014). The scope of BASE-II is to examine a broad range of aging-related variables from different health domains including genetics, internal medicine, immunology, psychology, sociology, and economics. To assess brain age gap, we make use of our recently established normative classifiers that we trained on structural MRI data from 32,634 adults from the UK Biobank cohort (Jawinski, 2022). Our classifiers provide estimates for the tissue types gray matter, white matter, and combined gray and white matter. We have decided to take into account both gray and white matter segmentations, because brain aging has been shown to encompass biologically distinct patterns of change, with tissue-specific analyses possibly granting additional and biologically more meaningful insights (Smith et al., 2020). In line with this, our own previous analyses suggest that gray and white matter brain age gap are genetically correlated at *r*_*G*_ = 0.70 (*SE* = 0.018), indicating both shared and segregated biological mechanisms (Jawinski, 2022). As carried out by the majority of previous investigations, we also calculated a single “all-in-one” brain age estimate.

Brain age gap has not been examined in the BASE-II cohort before, which gives us the opportunity to assess the following three research questions: First, we seek to corroborate previously shown associations between brain age gap and physical health, mental health, lifestyle factors, as well as socioeconomic status. We regard this is highly relevant, because reproducibility and replicability are key principles for scientific progress and have been identified as major issues in the natural sciences (Begley and Ellis, 2012; Aarts et al., 2015; Baker, 2016). Second, we seek to add to the current literature on the link between brain age gap and cognitive functioning. And third, we test associations of brain age gap with aspects of an individual’s time horizon such as future time perspective.

## Materials and Methods

In the following sections, we report how we determined our sample size, all data exclusions, all preparation and mining, and provide details about the measures in the study (Simmons et al., 2012). All analysis scripts have been made publicly available on GitHub.^1^

### Sample

The study population consisted of older participants drawn from the Berlin Aging Study II (BASE-II; Bertram et al., 2014; Gerstorf et al., 2016). Participants were recruited from the greater metropolitan area of Berlin, Germany, through advertisements in newspapers and public transportation systems as well as through a participant pool at the Max Planck Institute for Human Development. In the years 2012–2014, participants completed a comprehensive 2-day assessment program that consisted of a medical anamnesis performed by a physician, psychosocial surveys, a cognitive test battery, and a variety of laboratory tests. Information were collected on vision, hearing, physical capacity, the cardiovascular system, the muscolo-skeletal system, the immune system, as well as nutrition, social activities, political preferences and personality. Details on the assessment domains and specific tests have been provided previously (Bertram et al., 2014; Gerstorf et al., 2016). Older participants in BASE-II ranged in age from 61 to 88 years (*n* = 1,591; mean = 70.1; *SD* = 3.7; 50.9% female; Düzel et al., 2019). About 55% of all subjects reported a regular intake of one up to four medications, with another 25% reporting an intake of more than four medications (Toepfer et al., 2019). Prevalent diseases included arterial hypertension (73%), hyperlipidemia (76%), hypercholesterolemia (64%), diabetes (12%), chronic kidney disease (17%), as well as coronary heart disease (8%; Rosada et al., 2020). About 17% reported a history of depression (Demuth et al., 2021). Education and self-rated health were higher when compared to the general population (Bertram et al., 2014). After completing the main assessment program, MRI-eligible participants were invited to a structural brain imaging session within an average time interval of 3.2 months. Of the total 345 participants with structural T1-weighted MRI data, we included 337 participants who provided data for at least one of the 27 defined criterion variables of interest (see section “Criterion Variables”). Two participants of the MRI sample were not included due to severe MRI motion artifacts. This resulted in *N* = 335 older adults (127 female, age range: 61–82 years, mean age = 70.5 years; *SD* = 3.8 years) eligible for brain age gap analyses. Subjects gave written informed consent and received an expense allowance. All procedures were conducted according to the Declaration of Helsinki. BASE-II was approved by the Ethics Committee of the Charité-Universitätsmedizin Berlin (approval number EA2/029/09), and by the Ethics Committee of the Max Planck Institute for Human Research. In addition, the MRI protocol was approved by the Ethics Committee of the German Society for Psychology (DGPs, GA Kühn 012013_6).

### Magnetic Resonance Imaging Data Acquisition

Magnetic resonance imaging (MRI) data were acquired on a 3-Tesla Siemens Magnetom Trio scanner (Erlangen, Germany) using a 32-channel head coil. Structural T1 images were obtained according to the ADNI protocol.^2^ We applied a three-dimensional T1-weighted magnetization prepared rapid gradient-echo sequence (MPRAGE) in the sagittal plane with 2,500 ms repetition time (TR), 4.77 ms echo time (TE), 1,100 ms inversion time (TI), 7° flip angle, 140 Hz/pixel bandwidth, 256 × 256 × 176 acquisition matrix, 1 × 1 × 1 mm voxel size, 10.9 ms echo spacing, of 9:20 min duration.

### Magnetic Resonance Imaging Preprocessing

T1 images were preprocessed using the voxel-based morphometry pipeline of CAT12 (r1364)^3^ for SPM12 (r7487) in MATLAB 2018b. In brief, preprocessing involved affine and DARTEL registration of brain images to a reference brain, segmentation into gray matter, white matter, and cerebrospinal fluid, correction for bias-field inhomogeneities, and modulation of segmentations to account for the amount of volume changes due to spatial registration. Processed images were smoothed by applying an 8 mm full-width-at-half-maximum (FWHM) Gaussian kernel with subsequent resampling to 8 mm^3^ voxel size.

### Age Estimation Models

Age estimation models were trained through supervised machine learning in a sample of *N* = 32,634 unrelated, white-British ancestry individuals of the UK Biobank cohort (age range: 45–80 years). A detailed description of the UK Biobank study design, participants and quality control (QC) methods has been published previously (Bycroft et al., 2018). We were granted access to the UK Biobank dataset through application number 42032. Details on our age estimation procedure have been provided previously (Jawinski, 2022), and were adapted from Franke et al. (2010). In short, we first derived gray and white matter brain images by applying the CAT12 voxel-based morphometry to structural T1-weighted MRI scans (equivalent to the procedure described in section “Magnetic Resonance Imaging Preprocessing”). Age estimation models were then trained in a 10-fold cross-validation manner with 100 repeats. Therefore, we randomly split the UK Biobank imaging sample into ten equally sized subsets. Nine subsets served as training sample to build a statistical model that predicts the true chronological age from MRI data. The prediction model was subsequently applied to the MRI data of the left-out test sample to derive brain-predicted age estimates. After the first model was trained and applied, the next subset served as test sample, while the other nine subsets were selected for model training. This procedure was carried on until each subset served as test sample. Models were trained separately on gray matter and white matter segmentations. Before applying the machine learning algorithms, we excluded voxels that did not show any variation across individuals. Moreover, we carried out principal component analyses (PCA) to remove redundant information and reduce dimensionality. The first 500 principal components served as feature set, which explained about 90% of the total variance observed across individuals in gray matter and white matter brain images, respectively. Age estimation models were trained using three different types of machine learning algorithms: We made use of the sparse Bayesian “Relevance Vector Machine” in MATLAB (Tipping, 2001), and we used the gradient boosting package “xgboost” v.0.82.1 in R with both the decision tree and linear booster (Chen et al., 2019). To improve prediction performance, age estimates derived from applying the three types of machine learning algorithms (relevance vector machine, xgboost with decision tree booster, and xgboost with linear booster) were stacked for each tissue type and across tissue types, respectively, by linear regression. This resulted in three brain-predicted age estimates per subject, representing the tissue types gray matter, white matter, and combined gray and white matter. Our age estimation models have previously achieved excellent prediction accuracies, with mean absolute errors (MAE) ranging between 3.09 and 3.37 years, and correlation coefficients between brain-predicted and chronological age ranging between *r* = 0.83 (*R*^2^ = 0.68) and *r* = 0.86 (*R*^2^ = 0.73) in the UK Biobank cohort (age range: 40–85 years, Jawinski, 2022). Prediction accuracies reached similar levels (combined gray and white matter: MAE = 3.56 years, *r* = 0.86) in an independent MRI sample of about 1,900 individuals (age range: 45-80 years) of the LIFE-Adult cohort (Loeffler et al., 2015; Engel et al., 2022).

### Brain Age Gap Calculation

Gray matter, white matter, and combined gray and white matter brain age gap estimates were calculated by subtracting the chronological age from the predicted age of an individual. Due to regression dilution and non-Gaussian distribution of subject ages (Smith et al., 2019), brain age gap estimates have commonly been observed to be confounded by age (i.e., younger participants’ ages are systematically overestimated and older participants’ ages are underestimated). In line with the previous literature, we removed this bias by using age and age^2^ as covariates in all association analyses (Kaufmann et al., 2019; Smith et al., 2019; Cole, 2020). Importantly, since the brain age gap paradigm draws inferences based on errors in model prediction, validation of brain age gap with other meaningful variables is essential. For the models applied in the present study, we have previously shown that brain age gap is under substantial genetic control, with about 30% of the phenotypic variance explained by common genetic variation (Jawinski, 2022). Further, we have demonstrated their excellent test-retest reliability (ICCs ranging from 0.88 to 0.92 with a 2-year test-retest interval) and external validity when correlated with other UK Biobank phenotypes such as “overall health ratings” and “fluid intelligence.”

### Criterion Variables

Criterion variables were selected based on the comprehensive review article by Franke and Gaser (2019), The review article puts particular emphasis on brain age gap estimates derived from applying voxel-based morphometry. It should be noted, though, that we did not explicitly include studies based on feature type or modality, and essentially regarded every brain MRI study that was set out to examine associations of brain-predicted age estimates as “brain age” study. For an overview of modalities, procedures, and feature sets used by the studies we refer to, please see Supplementary Table B1.

A large proportion of variables examined in the present study have first been linked to brain age gap by Franke et al. (2013, 2014). This concerns measures of drinking and smoking behavior, depression, diabetes-related and metabolic syndrome variables, as well as a variety of blood laboratory parameters. Moreover, we considered findings on mild cognitive impairment (Franke and Gaser, 2012; Gaser et al., 2013) and socioeconomic status (Steffener et al., 2016; Smith et al., 2019). Our investigation on cognitive performance variables and time horizon was based on Smith et al. (2019) and Düzel et al. (2016; 2018b, see also Murphy and Dockray, 2018), respectively. In the following, we provide a brief overview of all criterion variables employed in the present study. Please see section “Assessment of Criterion Variables” in Supplementary Material A for further details. A list of all criterion variables with hypothesized effect directions, references to previous articles which have provided support for an association with brain age gap, and a list of all MRI datasets employed by these previous studies, is shown in Supplementary Table B1.

In a first step, we sought to confirm previously shown associations between brain age gap and socioeconomic status, lifestyle factors, and variables related to mental and physical health. We considered years of education and monthly household net income as indicators of socioeconomic status, which have been assessed *via* the German Socio-Economic Panel (SOEP) questionnaire (Goebel et al., 2019). Furthermore, we considered smoking status as single-item variable and the three items of the Alcohol Use Disorder Identification Test-Consumption (AUDIT-C; Bush et al., 1998), measuring amount and frequency of alcohol intake as well as heavy episodic drinking (binge drinking), as indicators of lifestyle. Regarding mental health, we took into account results derived from the Mini-Mental-State-Examination (MMSE, Folstein et al., 1975), the Geriatric Depression Scale (GDS, Yesavage et al., 1982), and the CES-D screening test for depression (Lewinsohn et al., 1997). We also considered a variety of physical health variables including diabetes diagnosis, systolic and diastolic blood pressure, body mass index as well as the metabolic load factor, i.e., a latent factor score that represents the five major indicators of metabolic syndrome (Düzel et al., 2018a). Moreover, we took into account laboratory parameters including fasting blood glucose, post-load glucose and hemoglobin A1c (HbA1c) as diabetes-related criterion variables. We also calculated the Homeostasis Model Assessment insulin resistance (HOMA-IR) index, i.e., a marker that is predictive for metabolic syndrome (Gayoso-Diz et al., 2013). We also considered serum concentrations of gamma-glutamyl-transferase and uric acid, as well as tumor necrosis factor alpha (TNF-α). In a second step, we sought to add to the current literature on the associations between brain age gap and cognitive performance variables. We here focused on the digit symbol substitution test performance as a manifest score as well as extracted factor scores of working memory, episodic memory, and fluid intelligence (for a detailed description of the latent factor models see Supplementary Material in Düzel et al., 2016). Third, we tested novel potential associations with an individual’s time horizon including scores derived from the Future Time Perspective Scale (Carstensen and Lang, 1996) and Consideration of Future Consequences Scale (Strathman et al., 1994). In total, 27 criterion variables were tested for an association with brain age gap.

### Statistical Analyses

Statistical analyses were carried out using R version 4.04 (R Core Team, 2021) and MATLAB R2018a (The MathWorks Inc., Natick, Massachusetts, United States). We performed partial Pearson correlations between brain age gap and the 27 criterion variables adjusting for confounding effects of sex, age, age^2^, and total intracranial volume. We selected this set of confounds in accordance with our previous UK Biobank investigation, where each of these confounds has been shown to independently correlate with brain age gap (Jawinski, 2022). We hypothesized that higher brain age gap is related to overall less favorable health characteristics and behavior, e.g., higher depression scores, more frequent alcohol intake, higher risk for diabetes and mild cognitive impairment, as well as lower fluid intelligence. We formulated directed hypotheses for all criterion variables (see Supplementary Table B1) and report one-tailed levels of significance. The nominal level of significance was set at *p* < 0.05. In order to determine the study-wise level of significance, we calculated the effective number of independent tests based on the eigenvalues derived from the bivariate correlation matrices of all variables of interest. The effective number of independent tests was estimated using R package poolr (Cinar and Viechtbauer, 2020) with method “Li Ji.” For the 27 criterion variables, the effective number of independent tests was estimated at 22. For the 3 brain age gap variables, the effective number of independent tests was estimated at 2. Considering a total number of 2 by 22 independent tests, we set the threshold of significance after multiple-testing correction at *p* < 0.001 (≈ 0.05/44). In line with this, subsequent permutation-based analyses (as described below) revealed that there was a 4.9% chance (i.e., a family-wise error-rate of α = 0.049) to observe at least one association with *p* < 0.001 among all tested associations under the null hypothesis of no effect. Based on our formulated hypotheses, we will consider results for discussion that reach at least nominal significance (*p* < 0.05).

Furthermore, we investigated whether the observed pattern of associations provides overall stronger evidence than expected under the null hypothesis of no effect. In this regard, we considered the complete set of associations and tested for a general inflation of test statistics, which is a particularly powerful approach in scenarios with multiple true associations. We previously carried out a similar procedure using permutation-based quantile-quantile plots (Hensch et al., 2019; Jawinski et al., 2021). In order to test for “overall stronger effects than expected under the null,” we repeated our partial correlation analysis between the 3 brain age gap and the 27 criterion variables (as described above) after randomly shuffling the empirical data. Specifically, in our data matrix (rows: subjects, columns: 3 brain age gap and 27 criterion variables), we randomly shuffled the rows of the 3 brain age gap variables (altogether), while the other entries of the matrix (criterion variables) were kept constant. In this vein, we preserved the original correlations within the group of brain age gap variables and within the group of criterion variables, while the original correlations between the two groups of variables were eliminated. After data permutation, any observed correlation between brain age gap and the criterion variables can be considered to occur at random. It should be noted that, before data permutation, we calculated the residuals of all variables by regressing them on the covariates sex, age, age^2^, and total intracranial volume. Pearson correlations were then computed based on the residualized variables and degrees of freedom were adjusted accordingly. Data permutation was repeated 1 million times (i.e., 1 million permutations), which resulted in 1 million sets of “expected results” (i.e., each set comprised 3 × 27 correlations expected under the null). We chose to carry out 1 million permutations in order to derive reliable permutation-based *p*-values while keeping the computational burden within reasonable limits. Permutation-based *p*-values were derived by comparing our “observed results” (i.e., the original correlations) against the 1 million sets of “expected results,” as described in the following.

First, we counted all observed associations with hypothesis-consistent effect directions and determined the corresponding *p*-value as the proportion of sets of expected results showing the same or a larger number of associations with hypothesis-consistent effect directions (e.g., 7,000 out of 1 million sets show the same or a larger number of associations with hypothesis-consistent effect directions: *p* = 0.007). In the same vein, we counted all observed nominally significant associations and determined the corresponding *p*-value as the proportion of sets of expected results showing the same or a larger number of nominally significant associations. Next, we calculated the average of the observed correlation coefficients (*via* the inverse hyperbolic tangent function also known as Fisher’s z transformation) and determined the corresponding *p*-value as the proportion of sets of expected results showing the same or a larger average correlation coefficient. Before averaging, the scale of criterion variables with hypothesized negative associations was inverted, so that any positive correlation indicated an association in the hypothesized direction and vice versa. Ultimately, we created a permutation-based quantile-quantile plot to visually compare the distribution of observed *p*-values against the distribution of expected *p*-values under the null hypothesis. The extent of deviation was quantified by calculating the inflation factor λ over all observed associations. The inflation factor λ is defined as the observed median χ^2^ divided by the expected median of a χ^2^ distribution with one degree of freedom (i.e., 0.4549364 corresponding to *p* = 0.5). The inflation factor λ has most commonly been used in genome-wide association studies (Yang et al., 2011). In scenarios with large numbers of true effects among the tested associations, λ is expected to increase. Significance of λ was determined as the proportion of sets of expected results showing the same or a larger λ.

### Statistical Power

We carried out sensitivity power analyses (see Lakens, 2021) using r package pwr (Champely, 2020), with effect sizes quantified as Pearson’s rho. Across the 27 criterion variables, the number of observations varied between 160 and 335. Given *N* = 335 and α = 0.05, power calculations revealed that associations with true effect sizes of *r* = 0.044, *r* = 0.090, and *r* = 0.135 were identified with a chance of 20, 50, and 80% (1-β), respectively. Considering the variable with the lowest number of observations (*N* = 160), power calculations suggested that associations with true effect sizes of *r* = 0.064, *r* = 0.130, and *r* = 0.195 were identified with a chance of 20, 50, and 80% (1-β), respectively. Supplementary Figure A1 shows the probabilities of associations to reach the level of significance, given true effect sizes of up to *r* = 0.4.

## Results

### Prediction Accuracies

**FIGURE 1:**
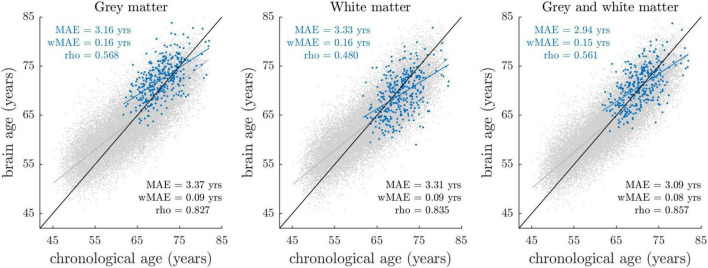
Brain-predicted (“brain age”) vs. chronological age stratified by sample and tissue class. Blue dots reflect the estimates of the BASE-II sample (*N* = 335), with their fitted linear regression line shown in blue. Gray dots reflect the estimates of the UK Biobank imaging cohort (*N* = 32,634), among whom age estimation models were trained and applied in a 10-fold cross-validation manner. The linear regression line fitted on the UK Biobank data is shown in gray. The identity line (y = x line) is shown in black. At this stage, brain-predicted age estimates have not been corrected for regression dilution, that is, the overestimation of younger participant’s ages and vice versa. Prediction accuracy (blue: BASE-II, black: UK Biobank) was quantified by MAE (mean absolute error between brain-predicted and chronological age), wMAE (weighted MAE defined as ratio between MAE and age range) and rho (Pearson’s correlation coefficient between brain-predicted and chronological age).

Figure 1 shows the distributions of gray matter, white matter, and combined gray and white matter brain age as a function of chronological age in BASE-II and UK Biobank cohort. The overlapping distributions of estimates in BASE-II and UK Biobank suggest overall good agreement of model performances.

Due to the different chronological age range in BASE-II (61–82 years) relative to UK Biobank (45–80 years), differences in model accuracy parameters mean absolute error (MAE) and rho (correlation coefficient between brain-predicted and chronological age) are an expected finding. To compare model accuracies across studies with different age ranges, the weighted mean absolute error (wMAE) as ratio of MAE and age range has been proposed (Cole et al., 2019). We observed lower wMAEs in BASE-II when compared to UK Biobank, suggesting lower model accuracies in BASE-II. Notably, wMAE has been reported to vary as a function of age range, as it does not account for the underlying age distribution (de Lange et al., 2022). Therefore, we additionally matched UK Biobank to BASE-II participants by chronological age and sex using R package MatchIt (Ho et al., 2011), and recalculated prediction accuracies for the resulting UK Biobank subset (for details see section “Comparison of Age Prediction Accuracies in BASE-II and UK Biobank” in Supplementary Material A). In this matched UK Biobank subset, correlations between brain-predicted and chronological age ranged between *r* = 0.62 and *r* = 0.66, with MAE ranging from 3.08 to 3.31 years. In BASE-II, correlations ranged between *r* = 0.48 and *r* = 0.57, with MAE ranging from 2.94 to 3.33 years. Hence, correlation coefficients suggested overall lower prediction accuracies in BASE-II. At the same time, we observed lower MAE that appeared to result from a general bias toward higher brain age estimates in BASE-II (Supplementary Figure A2). We argue that lower correlations and overall higher brain age estimates likely result from different MRI scanner properties and acquisition procedures, which we address in the discussion section of this article. In sum, prediction accuracy metrics suggested somewhat lower but still good model accuracies in BASE-II when compared to UK Biobank.

### Descriptive Statistics of Criterion Variables

Descriptive statistics of the 3 brain age gap and 27 criterion variables are shown in Table 1. First-order and partial correlation matrices as well es hierarchical cluster analysis results of the 27 criterion variables are shown in Supplementary Figures A3–5 (for intercorrelations of brain age gap variables see Supplementary Table A1).

**TABLE 1:** Descriptive statistics of the three brain age gap and 27 criterion variables.

	*N*	Mean	*SD*	Min	Max	Q1	Q2	Q3	Skew	Kurt
**Brain age gap**										
Gray matter (years)	335	0.00	2.99	−7.26	8.74	−1.89	0.08	2.06	−0.02	−0.19
White matter (years)	335	0.00	3.71	−12.07	9.49	−2.57	0.00	2.44	0.01	−0.08
Gray and white matter (years)	335	0.00	3.17	−9.03	8.15	−2.26	0.05	2.19	0.05	−0.29
**Replication**										
Years of education	300	14.05	2.88	7	18	12	13	18	0.14	−1.29
Household income (EUR)	221	2,376	1,259	430	10,000	1,600	2,200	2,800	2.09	8.32
Mini-mental state examination	326	28.52	1.49	18	30	28	29	30	−2.46	11.75
Geriatric depression scale	327	1.15	1.64	0	10	0	1	2	2.05	5.15
CES-Depression	327	6.42	5.94	0	31	2	5	9	1.51	2.29
Smoking status	278	“Never”: 134, “stopped more than a year ago”: 117, “stopped less than a year ago”: 3, “current smoker”: 24								
Frequency of alcohol intake	163	“Never”: 3, “once a month or less”: 30, “two to four times a month”: 42, “two to four times a week”: 40, “four times a week or more”: 48								
Amount of alcohol intake	160	“One to two glasses”: 122, “three to four glasses”: 32, “five to six glasses”: 6, “seven to nine glasses”: 0, “ten or more glasses”: 0								
Frequency of 6 glasses of alcohol intake	161	“Never”: 120, “less than once a month”: 36, “once a month”: 2, “once a week”: 3, “daily or almost daily”: 0								
Diabetes diagnosis	328	controls: 294, cases: 34								
HOMA-Insulin resistance	318	2.61	3.24	0.10	45.71	1.28	1.86	2.99	8.71	104.36
Hemoglobin A1c (%)	322	5.58	0.55	4.70	9.80	5.30	5.50	5.80	2.90	15.74
Fasting glucose (mg/dl)	294	96.41	21.18	67	241	86	91	100	3.45	16.93
Post-load glucose (mg/dl)	276	110.39	38.24	27	275	87	103	123	1.61	3.47
Body mass index (kg/m^2^)	327	26.69	3.46	18.59	40.16	24.29	26.56	28.93	0.39	0.47
Diastolic blood pressure (mmHg)	281	84.72	10.94	50	130	77	85	92	0.39	1.11
Systolic blood pressure (mmHg)	281	145.52	18.12	80	205	133	145	156	0.26	0.74
Metabolic load factor	321	0.01	0.14	−0.24	0.77	−0.08	−0.02	0.07	1.78	6.14
Gamma-glutamyl-transferase (U/L, serum)	327	30.08	29.08	6	273	16	22	33	4.84	30.49
Uric acid (mg/dL, serum)	327	5.48	1.28	2.60	9.60	4.60	5.50	6.20	0.40	0.46
Tumor necrosis factor-alpha (pg/ml)	307	0.82	3.48	0.00	47.74	0.00	0.14	0.44	10.30	124.15
**Cognition**										
Digit symbol substitution test	324	44.93	9.78	16	90	39	44	50	0.38	1.04
Episodic memory	335	0.03	0.34	−0.90	1.04	−0.20	0.05	0.26	−0.03	−0.13
Working memory	335	0.05	0.61	−1.39	2.19	−0.36	0.07	0.46	−0.01	0.11
Fluid intelligence	335	0.03	0.71	−1.52	2.45	−0.50	0.11	0.54	−0.05	−0.18
**Time horizon**										
Future time perspective	332	2.65	0.69	1.00	4.90	2.18	2.65	3.10	0.34	0.13
Consideration of future consequences	335	3.24	0.47	2.00	4.86	2.86	3.29	3.57	0.29	0.19

*SD, standard deviation; Min, minimum observed value; Max, maximum observed value; Q1, quartile 1; Q2, median; Q3, quartile 3; Skew, skewness; Kurt, excess kurtosis. Note that brain age gap variables were bias-corrected for sex, age, age^2^, and total intracranial volume. Variables “smoking status,” “frequency of alcohol intake,” “amount of alcohol intake,” “frequency of 6 glasses of alcohol intake,” and “diabetes diagnosis” were numerically coded to range from 0 to the number of respective categories minus 1 (e.g., 0–3 for smoking status).*

### Brain Age Gap Associations

**FIGURE 2:**
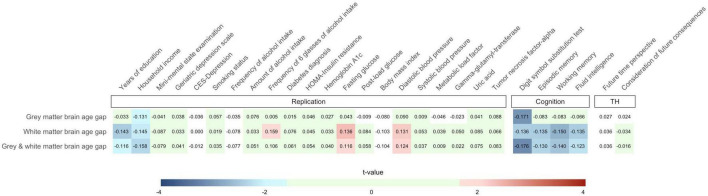
Partial Pearson correlations between the 27 criterion variables and gray matter, white matter, and combined gray and white matter brain age gap. Effects of sex, age, age^2^, and total intracranial volume were partialled out. Only cells containing associations with *p* < 0.05 (one-tailed) have been assigned with colors of the blue and red color palette. Green cells show associations not reaching nominal significance (*p* ≥ 0.05) but with hypothesis-consistent effect directions. Note that the number of observations varied across the criterion measures so that stronger associations do not necessarily reflect results with lower *p*-values. We provide an interactive version of this plot at https://github.com/pjawinski/base2/. TH, time horizon.

Partial Pearson correlations between brain age gap and the 27 criterion variables are summarized in Figure 2. We provide an interactive version of Figure 2 with additional information on GitHub. Detailed association results are provided in Supplementary Table A3.

#### Replication

For replication analyses, we considered twenty-one variables related to socioeconomic status, mental health, as well as physical health. Six variables were associated at *p* < 0.05. In particular, we observed that higher brain age gap is linked to fewer years of education (white matter: *r* = −0.143, *p* = 0.007; gray and white matter: *r* = −0.116, *p* = 0.023), lower household income (gray matter: *r* = −0.131, *p* = 0.027; white matter: *r* = −0.145, *p* = 0.016; gray and white matter: *r* = −0.158, *p* = 0.010), higher frequency of six glasses of alcohol intake (white matter: *r* = −0.159, *p* = 0.023), higher fasting blood glucose levels (white matter: *r* = 0.136, *p* = 0.010; gray and white matter: *r* = 0.116, *p* = 0.024), as well as higher diastolic blood pressure (white matter: *r* = 0.131, *p* = 0.015; gray and white matter: *r* = 0.124, *p* = 0.019).

#### Cognition

All four cognitive domains showed significant associations with brain age gap at the level of nominal significance. In particular, higher brain age gap significantly correlated with lower performance in the digit symbol substitution test (gray matter: *r* = −0.171, *p* = 0.001; white matter: *r* = −0.136, *p* = 0.007; gray and white matter: *r* = −0.176, *p* = 8E-4), lower episodic memory capacity (white matter: *r* = −0.135, *p* = 0.007; gray and white matter: *r* = −0.130, *p* = 0.009), lower working memory capacity (white matter: *r* = −0.150, *p* = 0.003; gray and white matter: *r* = −0.140, *p* = 0.005), as well as lower fluid intelligence (white matter: *r* = −0.135, *p* = 0.007; gray and white matter: *r* = −0.123, *p* = 0.013). The observed link between the digit symbol substitution test performance and combined gray and white matter brain age gap (*r* = −0.176, *p* = 8E-4) reached the level of significance after stringent correction for multiple testing.

#### Time Horizon

Moreover, we carried out association analyses between brain age gap and variables of an individual’s time horizon including future time perspective and consideration of future consequences. None of the tested associations reached the level of significance (all *p* ≥ 0.269).

### Evidence for Overall Stronger Effects Than Expected Under the Null Hypothesis

Next, we conducted a series of permutation-based analyses in order to examine whether the observed associations provide overall stronger evidence than expected by chance. We observed that 19 out of 27 (*p* = 0.101), 23 out of 27 (*p* = 0.006), and again 23 out of 27 (*p* = 0.006) tested associations, respectively, showed hypothesis-consistent effect directions for gray matter, white matter, and combined gray and white matter brain age gap (*p*-values derived from 1 million permutations). In total, across the three brain age gap variables, 65 out of 81 tested associations (80%) showed effects in the expected direction (*p* = 0.002), and 19 out of 81 (23%, *p* = 0.001) reached nominal significance. Moreover, the observed mean correlations between brain age gap and the 27 criterion variables aggregated to *r* = 0.032 (*p* = 0.044), *r* = 0.066 (*p* = 2E-4), and *r* = 0.060 (*p* = 6E-4), respectively, and thus provided further support for an association of brain age gap with the set of criterion variables. The observed mean correlation across all 81 tests reached *r* = 0.053 (*p* = 0.001).

**FIGURE 3:**
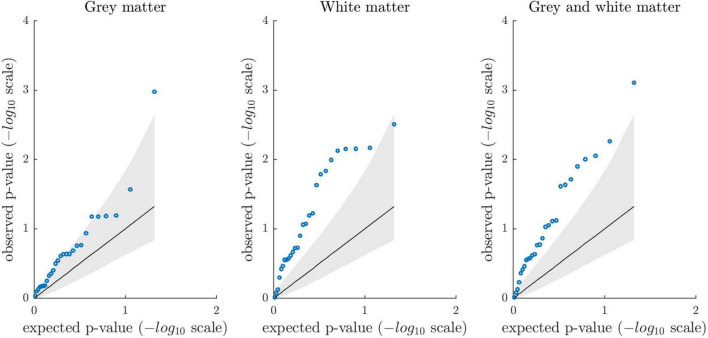
Permutation-based quantile-quantile plots showing the distribution of observed *p*-values from the association analyses (blue circles) sorted from largest to smallest and plotted against the expected *p*-values under the null hypothesis (1 million permutations; one-tailed testing). The solid diagonal line reflects the mean expected *p*-values (-log10 scale). The lower and upper bound of the gray shaded area represent the 5th and 95th percentile of the expected *p*-values. The plots show the association results between the 27 criterion variables and gray matter, white matter, and combined gray and white matter brain age gap, respectively. Overall, quantile-quantile plots suggest that association analyses revealed stronger evidence than expected under the null.

In addition, we created permutation-based quantile-quantile plots to compare the distribution of observed *p*-values against the distribution of expected *p*-values under the null hypothesis. Quantile-quantile plots indicated an excess of low *p*-values when compared to what would be expected by chance (Figure 3). To quantify the extent to which the distribution of observed *p*-values deviated from a random uniform distribution, we calculated λ over all observed associations (defined as median χ^2^/0.4549) and compared it against λ derived from the association results after 1 million permutations. The observed λ values were 2.94 (*p* = 0.028), 5.15 (*p* = 7E-4), and 4.16 (*p* = 0.004). Please see Supplementary Figure A6 for a quantile-quantile plot across all 81 tests (λ = 3.52, *p* = 0.005).

### Exploratory Analysis

Association analysis between brain age gap and the employed covariates indicated that age^2^ is not independently associated with any of the three brain age gap variables (Supplementary Table A2). In order to examine if our choice to include age^2^ as covariate has influenced the present statistical results, we repeated all partial correlation analyses between the 3 brain age gap and the 27 criterion variables without age^2^ serving as covariate. This resulted in virtually identical results with modest deviations of the observed *t*-statistics (Supplementary Figure A7). Nevertheless, concerning gray matter brain age gap, we observed two additional associations reaching nominal significance (episodic memory: *r* = −0.095, *p* = 0.041; working memory: *r* = −0.093, *p* = 0.045) and one additional association reaching the study-wise level of significance (digit symbol substitution test: *r* = −0.174, *p* = 9E-4). All other associations supported the same inferential decisions. Signs of all correlation coefficients remained unchanged. Results are shown in Supplementary Table A4, which is provided along with other *post hoc* analyses in Supplementary Material A, section “Exploratory Analyses.”

## Discussion

Individual deviations from normative aging are a key interest in aging research in order to understand different aging trajectories and to inform intervention strategies toward preserving physical and cognitive health in old age. Here, we estimated the biological age of the brain in participants of the Berlin Aging Study-II by applying a normative machine-learning model that was trained on 32,634 MRI scans from the UK Biobank cohort. We assessed the relationship between brain age gap, i.e., the difference between an individual’s brain-predicted and chronological age, and a total of 27 health-related criterion variables. Our analyses revealed overall stronger evidence for an association between higher brain age gap and less favorable health characteristics than expected under the null hypothesis of no effect. In particular, 80% of the tested associations showed hypothesis-consistent effect directions (50% expected under the null) and 23% reached nominal significance (5% expected under the null). A large proportion of significant brain age gap associations emerged from a cluster of variables covering both socioeconomic and cognitive performance measures (see Supplementary Figure A5 for cluster analysis results). However, individual associations showed typically weak effect sizes, and only one association survived a stringent correction for multiple testing, that is, the link between combined gray and white matter brain age gap and the digit symbol substitution test.

In the present study, a number of previously reported associations between brain age gap and health-related variables replicated at the level of nominal significance. First, we observed higher brain age gap to be linked to fewer years of education and lower household income (i.e., socioeconomic variables), which is consistent with the findings of Steffener et al. (2016) and Smith et al. (2019). A relationship between income and health is well-documented in the literature (Case et al., 2002) and might be traced back to better access to care in high-income households and a higher frequency of stressful situations in low-income households, which is detrimental to health. Similarly, educational attainment has commonly been found to be linked to better health, which has been attributed to economic and psychological resources (e.g., coping skills) as well as health-oriented behavior (Ross and Chia-Ling, 1995). Interestingly, aside from cross-sectional associations, evidence from a recent longitudinal study questions the view that educational attainment has beneficial effects on the speed of aging (Nyberg et al., 2021). Although we regard associations of brain age gap with educational attainment and household income as plausible findings, future studies may further elaborate their potential bidirectional interactions over the course of life.

As mentioned above, we observed the two socioeconomic variables (years of education and household income) to correlate not only with each other, but also with cognitive performance variables. In line with this, hierarchical cluster analyses suggested these variables to group together (Supplementary Figure A5). This is consistent with the previous use of educational attainment as proxy phenotype for cognitive performance (e.g., Okbay et al., 2016). Intriguingly, a large proportion of significant brain age gap associations in the present study emerged from this cluster, and due to the observed result consistency as well as one association reaching the study-wise level of significance, we regard our findings in this domain as particularly compelling. In addition, exploratory regression analyses (Supplementary Material A) suggested that the two socioeconomic variables independently correlate with brain age gap, which further strengthens the evidence for a link between this cluster of variables and brain aging.

Further, our results corroborate that higher brain age gap relates to alcohol consumption (Franke et al., 2013; Smith et al., 2019; Ning et al., 2020), higher blood pressure (Franke et al., 2014; Smith et al., 2019; Cole, 2020), and higher fasting blood glucose levels (Franke et al., 2013). None of the other metabolic syndrome or diabetes-related variables showed significant associations. Noteworthy, we considered higher body mass index as less favorable health characteristic and expected a positive association with brain age gap, which is line with the results of Franke et al. (2014). However, the present results rather point toward in inverse relationship which would be consistent with findings by Smith et al. (2019), who have demonstrated that lower body mass index is among the strongest correlates of higher brain age gap in the UK Biobank cohort. In general, the relationships between body mass index, brain atrophy, and cognitive function remains an ongoing debate (cf. Ronan et al., 2016) and requires further elaboration.

The current study consistently linked higher brain age gap to poorer performance in cognitive measures. Although an inverse relationship between brain age gap and cognitive performance has previously been suggested, reported associations have so far been domain-specific while the exact domains have not been unequivocally confirmed (Boyle et al., 2021). It is therefore of note that we found consistent associations across cognitive domains and across brain age gap estimates derived from different tissue classes. Our results are in line with the well-replicated association between brain age gap and processing speed (Richard et al., 2018; Boyle et al., 2021), and provide additional evidence for the link between brain age gap and working memory, episodic memory, and fluid intelligence.

Furthermore, we tested associations between brain age gap and aspects of an individual’s time horizon including future time perspective and consideration of future consequences. Future time perspective has previously been linked to better episodic memory (Düzel et al., 2016) as well as to differences in specific gray matter regions of the brain (Düzel et al., 2018b). In addition, the consideration of future consequences has been shown to correlate with illness preventive and health promotive behavior (Murphy and Dockray, 2018). Despite these previous indications, our analysis did not reveal convincing support for an association with brain age gap. Studies employing larger and more diverse samples might be needed to establish a link between brain age gap and aspects of an individual’s time horizon.

As mentioned above, we observed more hypothesis-consistent effect directions and more nominally significant associations than expected under the null, although only one association survived a stringent correction for multiple testing. These results may indicate that the true effects of bivariate associations are generally weak, and the current study’s statistical power was too low to identify individual associations reliably. Essentially, subtle associations are in line with previous estimates derived from large samples (Smith et al., 2019; Cole, 2020). In fact, the majority of top associations reported by Smith et al. (2019) showed effects beneath *r* = 0.1 in the UK Biobank cohort. Moreover, the median reported effect size across studies that support our current hypotheses (Supplementary Table B1) aggregates to *r* = 0.09. In the present study, the probability to identify a true effect of *r* = 0.09 at *p* < 0.05 reached about 50% (Supplementary Figure A1). The achieved statistical power to identify individual associations may thus be regarded as limited. In scenarios with multiple true associations, considering the whole set of observed test statistics may provide a more powerful approach to unravel subtle patterns of associations. By quantifying the inflation of test-statistics in the present dataset, we found that the overall evidence derived from the present statistical analyses is stronger than expected by random chance. We thus anticipate several true associations at subthreshold significance-levels in the present study and presume that more of them will reach significance in future studies with higher statistical power.

In addition to deriving a single “all-in-one” brain age estimate, we here investigated tissue-specific effects for gray and white matter. In comparison, the majority of previous voxel-based morphometry studies used gray matter segmentations only (e.g., Franke et al., 2010, 2014; Gaser et al., 2013; Koutsouleris et al., 2014) or combinations of gray and white matter (e.g., Franke and Gaser, 2012; Gaser et al., 2013; Cole et al., 2018). Some studies also used separate gray and white matter segmentations as well as other image types to derive brain age estimates (e.g., Cole et al., 2015; Jonsson et al., 2019). Noteworthy, multiple modes of brain age with distinct biological foundations have previously been proposed (Smith et al., 2020), and these have been argued to possibly grant biologically more meaningful insights when compared to an aggregated all-in-one measure. In line with this, our own previous work suggests both a shared and segregated genetic architecture of gray and white matter brain age gap (Jawinski, 2022). On these grounds, we regard the investigation of tissue-specific effects as logical and encouraging extension of previous works.

In the present study, we observed more convincing results for white matter relative to gray matter brain age gap, as indicated by a larger number of nominally significant results and a stronger deviation of the observed *p*-value distribution from the null distribution. Nevertheless, we urge to interpret the observed differences between gray and white matter with caution, given that there is a substantial degree of uncertainty regarding the “true” underlying effect sizes. Bearing in mind the confidence intervals around each point estimate, we argue that associations do not appear very different between gray and white matter in the present study. This might be exemplified by our exploratory analyses, where we recalculated partial correlations without age^2^ serving as covariate and observed gray matter to gain upon white matter to some extent: Although point estimates remained virtually unchanged, there were two additional associations for gray matter brain age gap that surpassed the nominal (episodic memory and working memory) and one that surpassed the study-wise threshold of significance (digit symbol substitution test). In sum, we believe that the present findings do not suggest a substantial heterogeneity of associations between the 3 brain age gap phenotypes and the 27 criterion variables. We postulate that larger studies are needed to shed light on putative differential associations.

The current study results on brain age gap in different tissue classes are well in agreement with previous studies that served to derive our hypotheses. This is underscored by an excess of associations reaching nominal significance and the large proportion of results with hypothesis-consistent effect directions. Our results thereby corroborate previous findings and foster their credibility, generalizability, and validity. It should be emphasized that the majority of studies we refer to employed voxel-based morphometry (Supplementary Table B1, references). Of these, some reported brain age estimates for combined gray and white matter tissue segmentations (Franke and Gaser, 2012; Franke et al., 2013; Cole et al., 2018), while others reported separate brain age estimates for gray and white matter (Cole et al., 2015), or they used gray matter tissue segmentations only (Franke et al., 2010, 2014; Gaser et al., 2013; Koutsouleris et al., 2014). We also considered studies providing support from surface-based morphometry and subcortical segmentations (Steffener et al., 2016; Kaufmann et al., 2019; Ning et al., 2020), as well as studies pursuing multimodal approaches with structural, functional, and diffusion tensor imaging (Liem et al., 2017; Smith et al., 2019; Cole, 2020). Despite the fact that biological aging cannot be considered a homogenous process across different tissue classes, there are apparent intercorrelations between brain age gap estimates derived from different feature sets and modalities (Smith et al., 2020). The underlying biological mechanisms captured by different analysis procedure may therefore contribute to the consistency across studies.

To our knowledge, pre-trained and publicly available brain age models are still very scarce. One reason for this is that the employed models often contain data that allow partial reconstruction of the training dataset and, thus, they pose privacy issues that hamper data sharing among researchers (Hahn et al., 2022). Shielding individual privacy is a major challenge in the field of machine learning and artificial intelligence. Still, there are some pre-trained models that can be accessed publicly. One of them has been made available by the ENIGMA group and requires uploading Freesurfer output *via* a web-based interface (Han et al., 2021).^4^ Another pre-trained model has been made available by Cole and colleagues (Cole et al., 2018).^5^ Cole and colleagues’ analysis pipeline is very similar to our own approach, using voxel-based morphometry with gray matter and white matter segmentations plus cerebrospinal fluid. In the present study, we decided to use our own age estimation models as they have been trained on an exceptionally large dataset (32,634 individuals). Using our own models also enabled us to ensure that no research participants’ privacy or consent is compromised (as may have been the case with uploading the data). Another advantage to be noted is that using custom models—and thereby introducing some degree of methodological heterogeneity—further strengthens the robustness and generalizability of results derived in this field of research. Still, we believe that future research will tremendously benefit from establishing shareable age estimation models as proposed by Hahn et al. (2022), as they facilitate research in small datasets, promote consensus-building and increase interpretability of results.

Brain age gap has frequently been interpreted as accelerated or decelerated biological aging. In line with this notion, an accelerated progression of brain aging has been shown in mild cognitive impairment and Alzheimer’s disease by comparing follow-up and baseline assessment (Franke and Gaser, 2012). However, brain age gap may also reflect stable individual differences that emerge at an ontogenetically early period and are carried into old age (Vidal-Piñeiro et al., 2021). In general, researchers should be cautious to draw inferences about intraindividual variation (within-person differences) from interindividual variation (between-person differences; Molenaar, 2004; Schmiedek et al., 2020) Further longitudinal approaches are needed to clarify whether brain age gap captures constant, early formed brain characteristics carried into age, or rather differences in the speed of aging over lifespan.

A long-term goal of biological aging research is to develop tailored interventions based on biological instead of chronological age. The feasibility of an accurate real-time brain age estimation framework for use in routine clinical MRI examinations has only recently been shown (Wood et al., 2022). Indications on brain atrophy derived from such a screening tool may help to identify individuals with poor health outcomes and guide clinical decision-making. Nevertheless, the small effect sizes observed in the current and in previous investigations raise the questions how this evidence may inform personalized treatment strategies. In this regard, we believe that whole-brain age estimates may provide valuable indications for an individual’s overall health status with an emphasis on capturing neurological, psychological, and cognitive traits. However, follow-up examinations are required to identify particular patient needs. To take a further step toward individualized medicine, we believe that deriving cell, tissue, region, function and modality-specific age estimates may be an encouraging strategy to identify health domains that require particular care and guide protective interventions.

### Limitations

Several limitations to our study need to be addressed. First, BASE-II is a convenience sample comprising above-average healthy participants and our results may thus not generalize to more vulnerable groups of the population. Healthy samples also imply lower variances in health-related variables, so that observed effect sizes may be lower and statistical tests more conservative when compared to tests in samples representative for the general population. More heterogenous samples may facilitate the identification of mental and physical health variables affected by, or contributing to, brain age gap.

Further, our results may not generalize across other age groups, e.g., younger adults, among whom brain atrophy, cognitive decline and civilization diseases may be less prevalent. Several associations may even be speculated to be reversed in childhood and adolescence, where lower relative brain age could reflect delayed brain maturation and may therefore be associated with lower cognitive performance metrics. The number of brain age studies in younger cohorts is just increasing (Brouwer et al., 2020; Hong et al., 2020; Ball et al., 2021; Cropley et al., 2021; Hedderich et al., 2021), so that more evidence is to be expected in the upcoming years. Along this way, other biological aging indicators such as telomere length and DNA methylation status may additionally advance our understanding of aging processes and could aid to identify individuals that would benefit from interventions (Henje Blom et al., 2015; Vetter et al., 2019; Marini et al., 2020).

Moreover, we here applied a cross-sectional and entirely correlative design which precludes any inference on temporal ordering and causality. While it seems intuitive that higher brain age gap results from unhealthy lifestyle choices such as smoking habits or alcohol consumption and results in cognitive decline, our data cannot rule out other causal directions. While it has been shown that brain age gap can predict later cognitive decline in patients with mild cognitive impairment and Alzheimer’s disease (Franke and Gaser, 2012), there is also evidence that higher brain age gap in midlife is preceded by a decline in cognitive functioning from childhood onward (Elliott et al., 2019). The BASE-II cohort is followed in a longitudinal design and we hope to derive more conclusive evidence from within-person repeated assessments that track how study participants age.

A further limitation refers to the lower accuracies of the age estimation models in BASE-II when compared to UK Biobank. Specifically, gray matter models appeared to systematically overestimate participants’ ages (reflected by a positive shift of the regression intercept), although the correlation between brain-predicted and chronological age suggested that between-subject differences were captured very well. In comparison, white matter models only showed a modest trend toward overestimation, while the respective correlation coefficient suggested a more pronounced drop in capturing between-subject differences. While age estimation models generalize across scanning hardware and field strength (Franke and Gaser, 2012), prediction accuracies tend to be lower when training and test dataset come from different cohorts (Liem et al., 2017). For our own cross-validated UK Biobank models, we have previously shown high prediction accuracies when applied to a large external MRI dataset of 1,900 subjects of the LIFE-Adult cohort (Jawinski, 2022). Noteworthy, the UK Biobank, LIFE-Adult and BASE-II cohort studies acquired brain-images on 3T Siemens scanners with 32-channel head coils and MPRAGE sequences. However, the three cohort studies employed different scanning systems (Skyra, Verio, and Trio), with varying acquisition duration (5:20, 5:06, and 9:20 min), relaxation time (TR; 2,000, 2,300, and 2,500 ms), echo time (TE; 2.01, 2.98, 4.77 ms) and inversion time (TI; 880, 900, and 1,100 ms). We regard these acquisition differences as plausible reason for varying image properties that result in diverging prediction accuracies. There may also be other unknown, systematic sources of variation (biologically meaningful differences between the average UK Biobank and BASE-II participant) that may have affected prediction accuracies in BASE-II. Essentially, training the models on a more heterogenous dataset derived from different scanner sites with varying acquisition protocols and hardware will likely increase robustness and generalization performance in future investigations (Liem et al., 2017).

Another limitation refers to the fact that our age estimation models only consider structural T1-weighted MRI scans, while a number of previous investigations have shown that multimodal neuroimaging (e.g., combinations of structural MRI, resting-state and task-based functional MRI, as well as diffusion tensor imaging) may increase prediction accuracy (Liem et al., 2017; Cole, 2020; Smith et al., 2020). A drawback of achieving higher prediction accuracies through combination of different modalities might be that biologically meaningful associations could be diluted, as shown by Smith et al. (2020). Therefore, it appears to remain crucial to not regard brain aging as homogeneous process, but to identify joint and segregated components of structural and functional change across imaging modalities.

Our study also has the following strengths: As replication study, we here foster the credibility of previous brain age studies and provide evidence of generalizability and validity of associations with health-related variables. At the same time, we show that our previously established UK Biobank models accurately predict chronological age in the independent BASE-II cohort, which is a prerequisite to derive meaningful brain age gap associations. In addition to the common approach of deriving a single “all-in-one” brain age estimate, we here investigated tissue-specific effects for both gray and white matter, providing additional insights into biological aging mechanisms. Moreover, given that studies addressing the link between specific cognitive domains and brain age gap are scarce, we here report on four relevant cognitive domains implicated in aging processes. As a novel approach, we tested potential associations of brain age gap and aspects of an individual’s time horizon.

## Conclusion

The present results point toward multifaceted links between brain age gap and health-related variables. In particular, we observed a cluster of socioeconomic and cognitive performance variables to constitute convincing correlates of brain age gap, whose potential bidirectional trajectories may be within the scope of future investigations. In general, it should be noted that individual associations appear to be weak, indicating a need for large sample sizes to identify and quantify effects reliably. Deriving cell-type, tissue, region, and function-specific age estimates, and their latent factors, may be a fruitful strategy to identify health domains that require particular attention in clinical settings, and may thereby guide individualized protective interventions.

## Data Availability Statement

The data presented in this study are available upon request from the BASE-II office (https://www.base2.mpg.de). The data are not publicly available due to them containing information that could compromise research participant privacy/consent. Our statistical analysis code has been made publicly available on GitHub at https://github.com/pjawinski/base2. Requests to access these datasets should be directed to BASE-II office, https://www.base2.mpg.de.

## Ethics Statement

The studies involving human participants were reviewed and approved by the Ethics Committee of the Charité-Universitätsmedizin Berlin (approval number EA2/029/09), the Ethics Committee of the Max Planck Institute for Human Research, and the Ethics Committee of the German Society for Psychology (GA Kühn 012013_6). The patients/participants provided their written informed consent to participate in this study.

## Author Contributions

PJ, SM, and DG: conceptualization. PJ, SM, and CG: methodology. PJ: formal analysis and visualization. SM, DG, ID, ES-T, GW, DG, UL, and SK: resources. PJ, JD, and SD: data curation. PJ and SM: writing—original draft preparation. SM, DG, and SK: supervision. DG and SK: project administration. ID, ES-T, GW, DG, UL, and SK: funding acquisition. All authors: writing—review and editing.

## Conflict of Interest

The authors declare that the research was conducted in the absence of any commercial or financial relationships that could be construed as a potential conflict of interest.

## Publisher’s Note

All claims expressed in this article are solely those of the authors and do not necessarily represent those of their affiliated organizations, or those of the publisher, the editors and the reviewers. Any product that may be evaluated in this article, or claim that may be made by its manufacturer, is not guaranteed or endorsed by the publisher.

## Footnotes

1
https://github.com/pjawinski/base2

2
www.adni-info.org

3
http://dbm.neuro.uni-jena.de

4
https://www.photon-ai.com/enigma_brainage

5
https://github.com/james-cole/brainageR
