# Supplementary material for: Linking Brain Age Gap to Mental and Physical Health in the Berlin Aging Study II
Source: Front Aging Neurosci. 2022 Jul 22;14:791222. doi: 10.3389/fnagi.2022.791222 (PMC9355695; doi:10.3389/fnagi.2022.791222)
Supplement: Supplementary file 1 [file Data_Sheet_1.PDF]

Supplementary materials to:

Linking brain age gap to mental and physical health in the Berlin Aging Study II

Supplementary Methods

Assessment of criterion variables 2

Fig. A1 Power analysis results 5

Supplementary Results

Comparison of age prediction accuracies in BASE-II and UK Biobank 6

Fig. A2 Brain-predicted vs. chronological age in BASE-II and matched UKB subset 6

Fig. A3 Pearson correlations between the 27 criterion variables 7

Fig. A4 Partial Pearson correlations between the 27 criterion variables 7

Fig. A5 Cluster analysis results of partial Pearson correlations 8

Fig. A6 Permutation-based quantile-quantile plot 8

Table A1 Pearson correlations between the three brain age gap variables 9

Table A2 Hierarchical linear regression for brain age gap and covariates 9

Table A3 Partial correlations between brain age gap and the 27 criterion variables 10

Explorative Analysis

Associations of years of education and household income with brain age gap 12

Fig. A7 Partial correlations with age^2 vs. without age^2 serving as covariate 11

Fig. A8 Observed effect sizes vs. literature effect sizes 13

Table A4 Partial correlations (without age^2) between brain age gap and the 27 criterion variables ... 11

Table A5 Unique associations of years of education and household income with brain age gap 12

References 14

Please see **supplementary Table B1** (not included in this document) for a list of all criterion variables, references to previous articles which have provided support for an association with brain age gap, and respective hypothesized effect directions.

Supplementary Methods

Assessment of criterion variables

For an overview of the topics covered in BASE-II and details on the assessment program, please see Bertram et al. (2014). Please also see **supplementary Table B1** for a list of all criterion variables with hypothesized effect directions and references to previous articles that have provided support for an association with brain age gap.

Sociodemographic data

We measured years of education and the monthly household net income via the German Socio-Economic Panel (SOEP) questionnaire, which is a multidisciplinary household survey applied to approximately 30,000 adults in Germany, every year (Goebel et al., 2019). The SOEP questionnaire is used to collect data on more than 120 socio-economic and behavioral variables, which have been extensively tested for their reliability and validity in the framework of panel studies (Frick et al., 2007). Years of education was computed as the sum of years of schooling (no degree: 7 years; lower school degree: 9 years; intermediary school: 10 years; degree for a professional college: 12 years; high school degree: 13 years; other: 10 years) and years of occupational training (apprenticeship: 1.5 years; technical schools: 2 years; civil servants apprenticeship: 1.5 years; higher technical college: 3 years; university degree: 5 years). Monthly household net income was assessed by asking ‘If you take a look at the total income of all members of the household: How much is the monthly household net income today?’, with answers provided as Euros per month.

Mini-mental state examination

The Mini-Mental State Examination (MMSE; Folstein et al., 1975) is a widely used test of cognitive function among the elderly. It aims at identifying individuals with mild cognitive impairment and dementia. The MMSE includes tests of temporal and spatial orientation, memory, attention, visual-spatial skills, and language. The maximum score is 30, with scores of 25 and above most frequently interpreted as normal cognition. In clinical settings, the MMSE has been reported with a sensitivity of 79.8% and a specificity of 81.3% in identifying dementia (Mitchell, 2009). In BASE-II, the MMSE was applied by trained research assistants and medical personnel.

Geriatric depression scale

Participants completed the 15-item short-form of the Geriatric Depression scale (GDS, Yesavage et al., 1982), which has been designed as screening instrument to measure symptoms associated with depression in older persons. Participants are required to answer no or yes with regard to how they felt in the past week. Example items include ‘Do you feel that your life is empty?’ and ‘Are you basically satisfied with your life?’. Each item response indicates the presence (score 1) or absence (0) of a depressive symptom. The scale composite was calculated as the sum of all item responses, with higher sum scores indicating stronger depressiveness. The GDS has been observed with 81% sensitivity and 78% specificity when compared with depression diagnoses derived from semi-structured clinical interviews (Mitchell et al., 2010). In BASE-II, the GDS items have been read to the participants in the form of a structured clinical interview.

CES-Depression

Participants completed the 20-item Center for Epidemiological Studies-Depression (CES-D) scale (Lewinsohn et al., 1997), which has been designed to assess symptoms of depression experienced over the past week. The questionnaire addresses different facets of depression including mood, feelings of helplessness and hopelessness, feelings of guilt and worthlessness, psychomotor retardation, sleep disturbances, and loss of appetite. Example items are ‘I did not feel like eating; my appetite was poor’

and ‘I enjoyed life’. Participants are asked to provide their responses on a 4-point scale (‘rarely’, ‘some’, ‘occasionally’, ‘most or all of the time’), with item scores ranging from 0 to 3. The scale composite was calculated as the sum of the 20 item scores, with higher scores indicating stronger depressiveness. In BASE-II, CES-D items have been read to the participants in the form of a structured clinical interview.

Lifestyle variables

The BASE-II assessment program included the collection of lifestyle variables (as well as data on medical and family history and medication) during an anamnesis carried out by the Charité Geriatric Research Group. We here focused on the variables ‘smoking status’, ‘frequency of alcohol intake’, ‘amount of alcohol intake’, and ‘frequency of 6 glasses of alcohol intake’. Each of these variables was measured by a single item. Smoking status was measured by asking ‘Do you currently smoke?’ with answers provided on a 4-point scale (‘1 – Yes.’; ‘2 – No, I stopped less than a year ago.’; ‘3 – No, I stopped more than a year ago.’; ‘4 – No, I never smoked.’). We inverted and recoded this item scale so that item scores ranged from 0 to 3, with higher scores indicating higher proneness towards smoking. The three single-item variables on alcohol intake reflect the first three items of the World Health Organizations’s Alcohol Use Disorders Identification Test (Saunders et al., 1993), also known as short version AUDIT-C (consumption items; Bush et al., 1998). The AUDIT-C is intended to identify at-risk individuals with harmful and heavy episodic drinking (binge drinking). Frequency of alcohol intake was measured by asking ‘How often do you drink alcohol?’ with answers provided on a 5-point scale (‘0 – Never’; ‘1 – Once a month or less.’; ‘2 – Two to four times a month.’; ‘3 – Two to four times a week.’; ‘4 – Four times a week or more.’). Amount of alcohol intake was measured by asking ‘If you drink alcohol, how many glasses do you usually drink that day? One glass corresponds to 0.33 l of beer, 0.25 l of wine or sparkling wine, and 0.02 l of spirits.’, with answers provided on a 5-point scale (‘0 – One to two glasses.’; ‘1 – Three to four glasses.’; ‘2 – Five to six glasses.’; ‘3 – Seven to nine glasses.’; ‘4 – Ten or more glasses.’). Frequency of six glasses of alcohol was measured by asking ‘How often do you drink six or more glasses of alcohol on one occasion (e.g., at dinner, at a party, etc.)’, with answers provided on a 5-point scale (‘0 – Never.’; ‘1 – Less than once a month.’; ‘2 – Once a month’; ‘3 – Once a week.’; ‘4 – Daily or almost daily.’).

Diabetes diagnosis

Information on diabetes status was obtained through a medical anamnesis. If no information was available from the medical anamnesis, diabetes status was determined based on the oral glucose tolerance test (see paragraph *Laboratory tests and immunological data*) as well as medication intake. Participants were asked to bring their medication plan as well as the medication packets used on a regular basis.

Laboratory tests and immunological data

A large number of blood and urine laboratory parameters was collected as part of a 2-day study protocol designed to measure each participant’s objective and subjective health status. Blood and urine parameters were measured centrally in a commercial, certified laboratory (Labor 28 GmbH, Berlin). The study protocol included an oral glucose tolerance test (oGTT) that was carried out in accordance with the WHO guidelines (WHO, 2004). The oGTT is a standard procedure to test for diabetes and is used to measure how quickly glucose is cleared from the blood. First, blood samples were collected after 8 hours of fasting. Subsequently, 75g of glucose dissolved in 250-300 ml water were ingested by mouth and blood samples were again collected after 120 min. We here focused on the blood parameters fasting glucose (mg/dl), post-load glucose (mg/dl) and glycated hemoglobin A1c (HbA1c; %). Moreover, we calculated the homeostasis model assessment of insulin resistance (HOMA-IR) index, i.e., a surrogate marker for insulin resistance that is predictive for metabolic syndrome (Gayoso-Diz et al., 2013). HOMA-IR was calculated as fasting insulin (µU/mL) × fasting glucose (mmol/L) / 22.5. In addition, we considered gamma-glutamyl-transferase (U/L; serum), uric acid (mg/dL, serum), and tumor necrosis factor-alpha (TNF-α, pg/ml) as potential correlates of brain age gap. Gamma-glutamyl-transferase is

produced in high concentrations in the liver and has been linked to liver dysfunction, excessive alcohol intake, as well as metabolic and cardiovascular risk (Lee et al., 2007). Uric acid has been linked to kidney dysfunction as well as metabolic syndrome, and cardiovascular disease (Kim et al., 2010). TNF-α is an inflammatory cytokine that plays an important role in resistance to infection and cancers (Idriss and Naismith, 2000).

Blood pressure

Systolic and diastolic blood pressure (mmHg) were assessed in a seated position after participants had rested for five minutes. We used an electronic device to measure blood pressure (boso-medicus memory, Jungingen, Deutschland). The mean of two measurements (left and right arm) was used for analysis.

Body mass index (BMI)

An objective measure of Body Mass Index (BM) was derived by trained research assistants and medical personnel. Height (cm) and weight (kg) were assessed, and BMI was calculated as height (m) divided by weight (kg^2).

Metabolic load factor

The metabolic load factor is a continues latent variable that was derived by applying structural equation modelling on five metabolic syndrome indicators (Düzel et al., 2018). The five indicators have been underscored by the joint statement of the International Diabetes Federation Task Force (2009) and include waist circumference, triglycerides, systolic/diastolic blood pressure, fasting blood glucose, and high density lipoprotein. The metabolic load factor has been suggested as composite score that reflects the metabolic condition more comprehensively. Higher scores suggest a higher metabolic burden. The metabolic load factor has been shown to be associated with morbidity, physical health, and cognition (Düzel et al., 2018).

Episodic memory, Working memory, and Fluid intelligence

BASE-II participants were invited to two cognitive sessions of 3.5 h each. The exact interval between the two sessions was seven days. Participants underwent the cognitive battery in groups of 4 to 6 individuals, and were instructed according to a standardized protocol. The cognitive battery consisted of 27 tests and questionnaires. We here focused on the cognitive abilities episodic memory, working memory, and fluid intelligence. These abilities were extracted as latent factor scores. For a detailed description of the latent factor models, please see the supplemental material in Düzel et al. (2016). The manifest tasks indicating episodic memory included the Verbal Learning and Memory Test (Helmstaedter and Durwen, 1990), Face Profession Task (Schacter et al., 1994), and Scene Encoding Task (Düzel et al., 2018). Working memory was indicated by the Number-N-Back Task, Letter Updating Task, and Spatial Updating Task (Schmiedek et al., 2010). Fluid intelligence was indicated by the Figural Analogies Task, Letter Series Task, and Practical Problem Task (Lindenberger et al., 1993). The scores were calculated as the sum of correct responses (Verbal Learning and Memory Test, Number-N-Back Task, Letter Updating Task, Figure Analogies Task, Letter Series Task, Practical Problem Task), averaged percentages of correct placements (Spatial Updating Task) and hits minus false alarms (Face Profession Task, Scene Encoding Task) with higher scores indicating better performance. For details on the different tasks, see the supplemental material in Düzel et al. (2016).

Digit Symbol Task

The Digit Symbol Substation Test (DSST) was carried out as part of the BASE-II cognitive test battery (see paragraph *Episodic memory, Working memory, and Fluid intelligence*). The DSST is a neuropsychological test included in the Wechsler Adult Intelligence Scale (WAIS; Wechsler et al., 2008), and has been shown to be sensitive to cognitive dysfunction, dementia, depression, and age (Jaeger, 2018). The DSST consists of digit-symbol pairs followed by a list of digits. Participants are asked to

write down the corresponding symbol under each digit as fast as possible. We assessed the number of correctly assigned symbols within the allotted time (90 sec). Test sheets were enlarged by 100% to reduce perceptual and motor difficulties.

Future time perspective

Future time perspective was assessed using the 10-item Future Time Perspective (FTP) scale by Carstensen and Lang (1996). The FTP scale seeks to measure an individual' global perceptions of his or hers remaining time to live and the opportunities within that time (example item: 'I expect that I will set many new goals in the future'). Partipants made their ratings on a Likert scale ranging from 1 ('strongly disagree') to 5 ('strongly agree). The scale composite was calculated as the unweighted mean across all items, with higher scores indicating a more open–ended perspective. The FTP scale has been reported with acceptable internal consistency (Cronbach's α = .84), and its validity has been underscored by its correlations with depression (r = -.32) and negative affectivity (r = .18; Kooij et al., 2018).

Consideration of future consequences

Consideration of future consequences was assessed using the 12-item Consideration of Future Consequences (CFC) scale by Strathman and colleagues (1994). The CFC scale was designed to assess the extent to which an individual focuses on the long- and short-term implications of his or her own behavior (example item: 'I consider how things might be in the future, and try to influence those things with my day to day behavior'). Partipants made their ratings on a Likert scale ranging from 1 ('strongly disagree') to 5 ('strongly agree'). The scale composite was calculated as the unweighted mean across all items, with higher scores indicating a stronger tendency towards considering long-term consequences of one's own behavior. The CFC scale has been reported with acceptable internal consistency (Cronbach's α = .79), and its validity has been underscored by its correlations with personality trait conscientiousness (r = .29; Kooij et al., 2018).

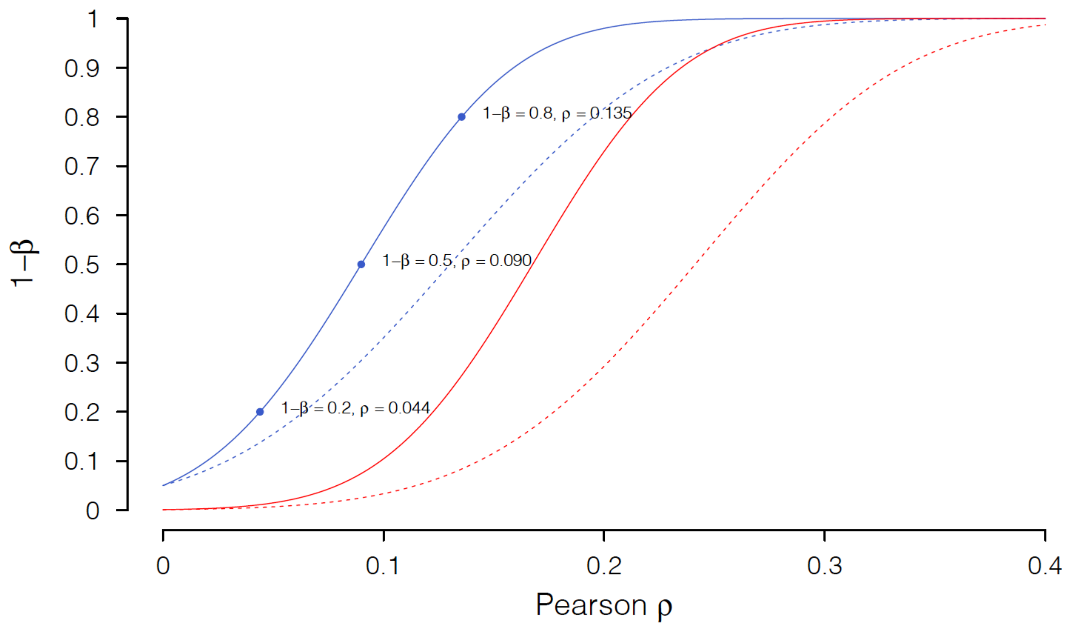

Fig. A1 Power analysis results showing the probability (1-β) of associations to reach the threshold of significance given true effect sizes ranging between ρ = 0.0 and ρ = 0.4. The blue curves show the probabilities of effects to reach nominal significance (α = 0.05, one-tailed) with $n = 335$ (solid curve) and $n = 160$ participants (dashed curve). The red curves show the probabilities of effects to reach the level of significance after multiple-testing correction (α = 0.001, one-tailed) with $n = 335$ (solid curve) and n = 160 participants (dashed curve). Power analysis was carried out using R package pwr v1.3-0 (Champely, 2020).

Supplementary Results

Comparison of age prediction accuracies in BASE-II and UK Biobank

Due to the different chronological age ranges in BASE-II and UK Biobank, differences in age prediction accuracy parameters MAE and rho are an expected finding. To adequately compare age prediction accuracies, we systematically matched UK Biobank participants to BASE–II participants by chronological age and sex. Matching was carried out using R package *MatchIt* (Ho et al., 2011) with distance measure *Mahalanobis* and ratio 10. Matching resulted in 3,350 UK Biobank participants (1270 female; age range: 61.75 – 81.86 years; mean age: 70.51 years) matched to 335 BASE-II participants (127 female; age range: 61.74 – 81.97 years; mean age: 70.52 years). The matched groups did not differ regarding sex (χ^2-test: $\chi^2 = 0$, $p = 1.00$) and age (t-test: $t_{3683} = 0.034$, $p = 0.973$, $d = 0.003$).

Figure A2 illustrates the prediction performances in BASE-II and the age- and sex-matched UK Biobank subset. In the matched UK Biobank subset, correlations between brain-predicted and chronological age ranged from $r = 0.62$ to $r = 0.66$, with mean absolute errors ranging from 3.08 to 3.31 years. In BASE-II, correlations ranged between $r = .48$ and $r = .57$, with mean absolute errors ranging from 2.94 to 3.33 years. Statistical comparisons suggested significantly lower correlation coeffcients in BASE-II with regard to white matter brain age gap (Fisher's z-test; $z = 3.402$, $p =$ 7E-4) and combined grey and white matter brain age gap (Fisher's z-test: $z = 2.586$, $p = 0.001$), but not with respect to grey matter brain age gap (Fisher's z-test; $z = 1.528$, $p = 0.127$).

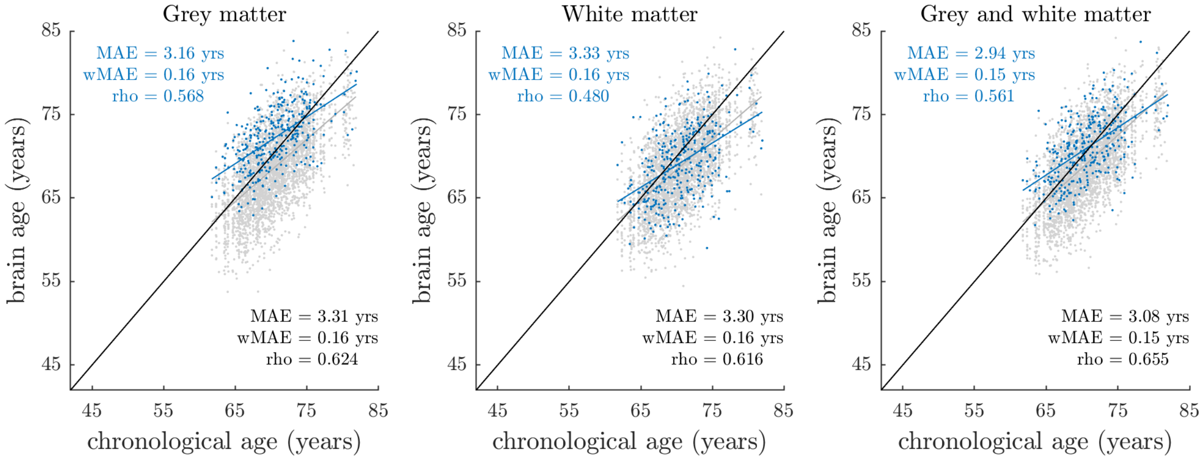

Fig. A2 Brain-predicted ('brain age') vs. chronological age stratified by sample and tissue class. Blue dots reflect the estimates of the BASE-II sample ($N = 335$), with their fitted linear regression line shown in blue. Grey dots reflect the estimates of the UK Biobank imaging cohort ($N = 32{,}634$), among whom age estimation models were trained and applied in a tenfold cross-validation manner. The linear regression line fitted on the UK Biobank data is shown in grey. The identity line (y=x line) is shown in black. Depicted brain-predicted age estimates have not been corrected for regression dilution, that is, the overestimation of younger particpant's ages and vice versa. Prediction accuracy (blue: BASE-II, black: UK Biobank) was quantified by MAE (mean absolute error between brain-predicted and chronological age), wMAE (weighted MAE defined as ratio between MAE and age range) and rho (Pearson's correlation coefficient between brain-predicted and chronological age).

From a descriptive point of view, we also observed overall lower mean absolute errors in BASE-II, which may suggest higher prediction accuracies. However, due to regression dilution, older participants' ages are typically underestimated. When compared to the matched UK Biobank subset, we observed a general trend towards higher brain age estimates in BASE-II with regard to grey matter (t-test: $t_{3683} = 13.764$, $p =$ 5E-42, $d = 0.789$), white matter (t-test: $t_{3683} = 1.743$, $p = 0.081$, $d = 0.100$), and combined grey and white matter (t-test: $t_{3683} = 7.348$, $p =$ 2E-13, $d = 0.421$). The overall lower mean absolute errors may thus be attributed to a general trend towards relatively higher brain age estimates in BASE-II. Higher brain age estimates in BASE-II may result from different MRI scanner properties and acquisition procedures, which we address in the discussion section of the main article.

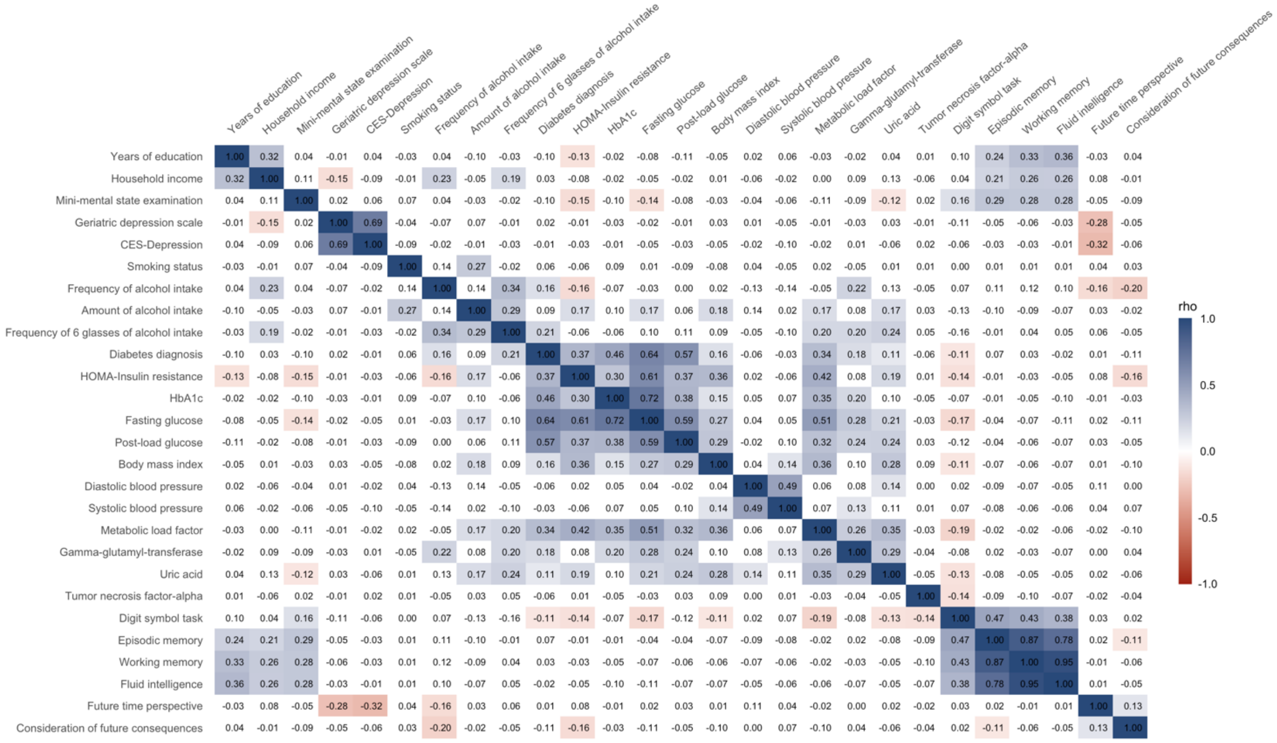

Fig. A3 Pearson correlations between the 27 criterion variables. Only cells with correlations reaching nominal significance ($p < 0.05$) have been assigned with colors of the blue and red color palette. For an interactive version of this plot including p-values and pair-wise sample sizes, please see https://github.com/pjawinski/base2.

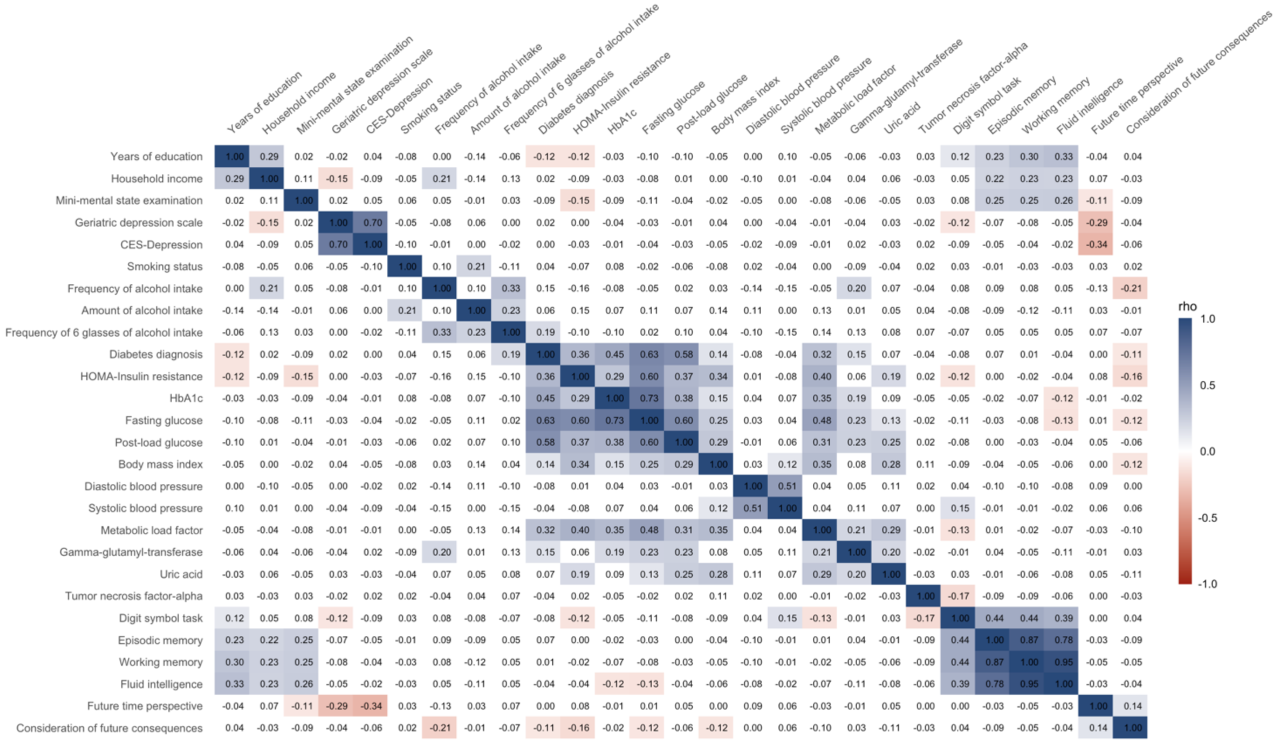

Fig. A4 Partial Pearson correlations between the 27 criterion variables. Sex, age, age^2, and total intracranial volume served as covariates. Only cells with correlations reaching nominal significance ($p < 0.05$) have been assigned with colors of the blue and red color palette. For an interactive version of this plot including p-values and pair-wise sample sizes, please see https://github.com/pjawinski/base2.

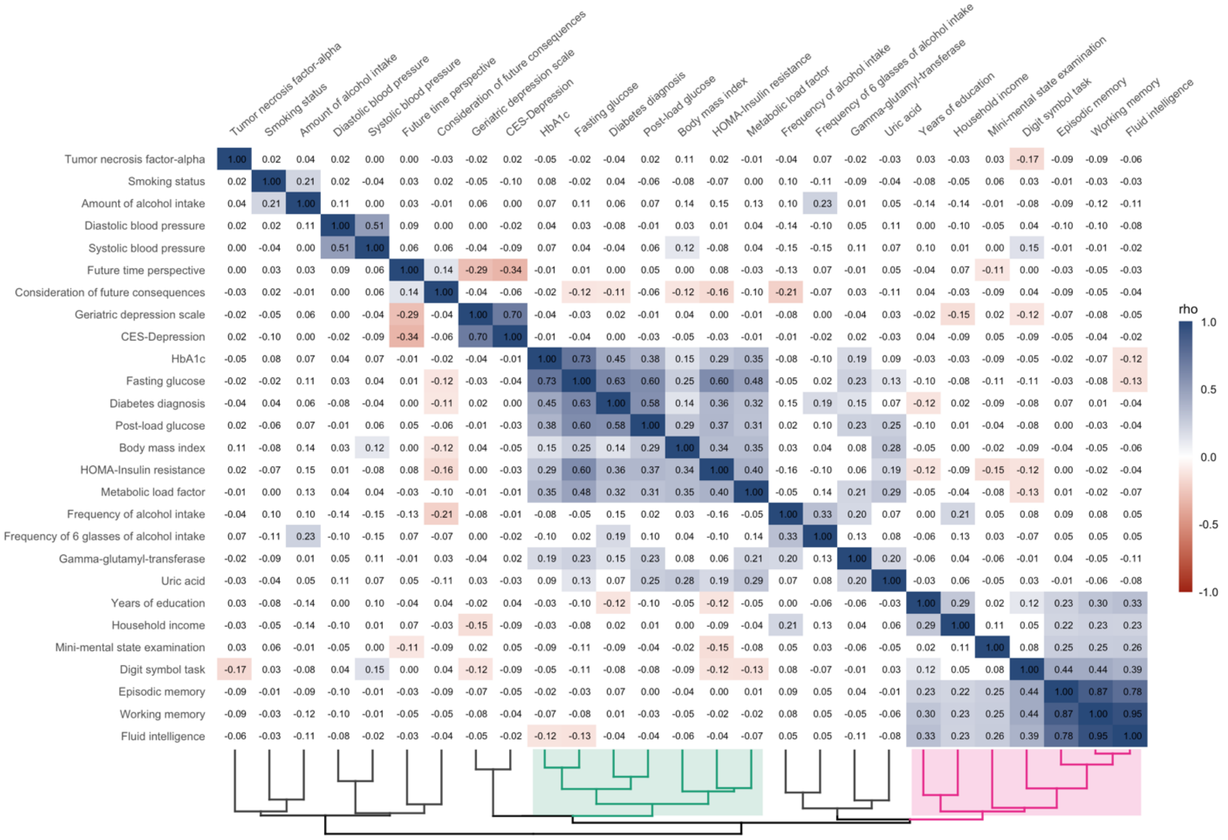

Fig. A5 Partial Pearson correlations between the 27 criterion variables (same results as shown in Fig. A4) hierarchically clustered using R function hclust with agglomeration method ‘complete’. Cluster analysis revealed two higher-order clusters with significant intercorrelations. Green: Cluster of diabetes and metabolic syndrome-related variables, Violet: Cluster of socioeconomic and cognitive performance variables.

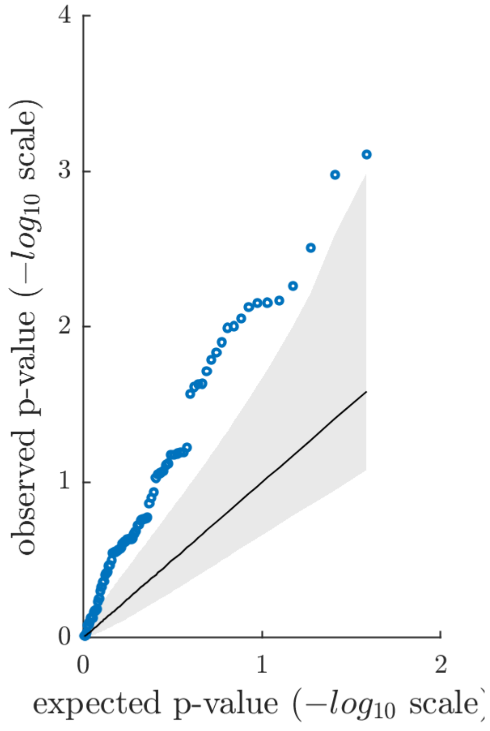

Fig. A6 Permutation-based quantile-quantile plot showing the distribution of observed p-values from the association analyses (blue circles) sorted from largest to smallest and plotted against the expected p-values under the null hypothesis (1 million permutations, one-tailed testing). The solid diagonal line reflects the mean expected p-values (-log10 scale). The lower and upper bound of the grey shaded area represent the 5th and 95th percentile of the expected p-values. The plot shows results of 81 tested associations (3 brain age gap variables by 27 criterion variables). Overall, the quantile-quantile plot suggests that association analyses revealed stronger evidence than expected under the null hypothesis of no effect.

Table A1 Intercorrelations between the three brain age gap variables

$N = 335$	Grey matter		White matter		Grey and white matter	
	r	*p*	rho	*p*	rho	*p*
Grey matter	1.000	-				
White matter	.545	6E-27*	1.000	-		
Grey and white matter	.828	2E-84*	.918	4E-134*	1.000	-

Results show partial Pearson correlations with sex, age, age^2, and total intracranial volume serving as covariates.

* $p < .05$ (two-tailed nominal significance)

Table A2 Hierarchical linear regression analysis for brain age gap and covariates

	Grey matter					White matter					Grey and white matter				
	R^2	dfn	dfd	*F*	*p*	R^2	dfn	dfd	*F*	*p*	R^2	dfn	dfd	*F*	*p*
Sex	0.040	333	1	14.0	2E-4 **	0.000	333	1	0.1	.763	0.010	333	1	3.2	.074
Sex, age	0.270	332	1	106.0	1E-21 **	0.190	332	1	77.3	8E-17 **	0.221	332	1	90.4	4E-19 **
Sex, age, TIV	0.282	331	1	5.7	.018 *	0.190	331	1	0.1	.761	0.225	331	1	1.7	.191
Sex, age, age2, TIV	0.285	330	1	1.6	.214	0.190	330	1	0.1	.711	0.227	330	1	0.8	.370

Note: The first row reflects simple linear regression results for brain age gap regressed on sex. All other rows show results of the hierarchical linear regression analysis that compares the model of the respective row (e.g., second row with sex and age used as regressors) against the model of the preceding row (e.g., sex used as regressor). Analysis was carried out with $N = 335$ subjects. grey matter: grey matter brain age gap, white matter: white matter brain age gap, grey and white matter: combined grey and white matter brain age gap, R^2: variance explained by the model, dfn: numerator degrees of freedom, dfd: denominator degrees of freedom, *F*: *F*-statistic, *p*: *p*–value

* p < .050
** p < .001

Table A3 Partial Pearson correlations between brain age gap and the 27 criterion variables

			Grey matter			White matter			Grey & white matter		
	h1	*n*	*r*	*t*	*p*	*r*	*t*	*p*	*r*	*t*	*p*
**Replication**											
Years of education	−	300	-.033	-0.569	.285	-.143	-2.483	.007 *	-.116	-2.000	.023 *
Household income	−	221	-.131	-1.936	.027 *	-.145	-2.150	.016 *	-.158	-2.347	.010 *
Mini-mental state examination	−	326	-.041	-0.733	.232	-.087	-1.559	.060	-.079	-1.425	.078
Geriatric depression scale	+	327	.038	0.682	.248	.033	0.589	.278	.041	0.728	.234
CES-Depression	+	327	-.080	-1.446	.925	-.022	-0.397	.654	-.053	-0.943	.827
Smoking status	+	278	.057	0.934	.176	.019	0.310	.378	.035	0.576	.282
Frequency of alcohol intake	+	163	-.035	-0.442	.670	-.078	-0.983	.836	-.077	-0.967	.832
Amount of alcohol intake	+	160	.076	0.950	.172	.033	0.410	.341	.051	0.634	.264
Frequency of 6 glasses of alcohol intake	+	161	.005	0.068	.473	.159	2.004	.023 *	.106	1.323	.094
Diabetes diagnosis	+	328	.015	0.265	.395	.076	1.361	.087	.061	1.095	.137
HOMA-Insulin resistance	+	318	.046	0.822	.206	.045	0.789	.215	.054	0.949	.172
Hemoglobin A1c	+	322	.027	0.479	.316	.033	0.583	.280	.040	0.703	.241
Fasting glucose	+	294	.043	0.736	.231	.136	2.333	.010 *	.116	1.979	.024 *
Post-load glucose	+	276	-.009	-0.151	.560	.084	1.378	.085	.058	0.960	.169
Body mass index	+	327	-.080	-1.442	.925	-.103	-1.852	.968	-.104	-1.878	.969
Diastolic blood pressure	+	281	.090	1.506	.067	.131	2.191	.015 *	.124	2.078	.019 *
Systolic blood pressure	+	281	.009	0.154	.439	.053	0.881	.189	.037	0.610	.271
Metabolic load factor	+	321	-.046	-0.815	.792	.039	0.698	.243	.009	0.165	.434
Gamma-glutamyltransferase	+	327	-.023	-0.404	.657	.050	0.888	.187	.022	0.402	.344
Uric acid	+	327	.041	0.736	.231	.085	1.522	.064	.075	1.350	.089
Tumor necrosis factor-alpha	+	307	.088	1.525	.064	.066	1.148	.126	.083	1.438	.076
**Cognition**											
Digit symbol substitution test	−	324	-.171	-3.100	.001 *	-.136	-2.448	.007 *	-.176	-3.191	8E-4 **
Episodic memory	−	335	-.083	-1.504	.067	-.135	-2.468	.007 *	-.130	-2.383	.009 *
Working memory	−	335	-.083	-1.514	.065	-.150	-2.755	.003 *	-.140	-2.559	.005 *
Fluid intelligence	−	335	-.066	-1.200	.115	-.135	-2.471	.007 *	-.123	-2.249	.013 *
**Life perspective**											
Future time perspective	-	332	.027	0.482	.685	.036	0.659	.745	.036	0.656	.744
Consideration of future consequences	−	335	.024	0.429	.666	-.034	-0.617	.269	-.016	-0.288	.387

Partial Pearson correlations were carried out with sex, age, age^2, and total intracranial volume serving as covariates. As we used four covariates, all t-values are specified by n-2-4 degrees of freedom.

h1: hypothesized effect direction (positive + or negative − association with brain age gap), grey matter: grey matter brain age gap, white matter: white matter brain age gap, grey & white matter: combined grey and white matter brain age gap, *n*: number of observations, *r*: Pearson's correlation coefficient, *t*: *t*-statistic, *p*: *p*–value (one-tailed)

* $p < .05$ (one-tailed nominal significance)
** $p < .001$ (one-tailed significance after multiple testing correction)

Exploratory Analyses

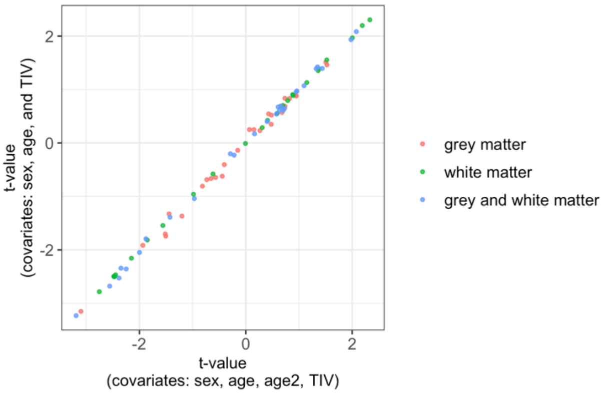

Fig. A7 Comparison of partial Pearson correlation results (t-values) with age^2 serving as covariate (x-axis) vs. without age^2 serving as covariate (y-axis). Correlations were calculated between brain age gap variables (grey matter, white matter, and combined grey and white matter) and the 27 criterion variables. The plot indicates that association results were not substantially altered by partialling out effects of age^2. TIV: total intracranial volume.

Table A4 Partial Pearson correlations (not controlling for age^2) between brain age gap and the 27 criterion variables

			Grey matter			White matter			Grey & white matter		
	h1	*n*	*r*	*t*	*p*	*r*	*t*	*p*	*r*	*t*	*p*
**Replication**											
Years of education	-	300	-.038	-0.646	.259	-.144	-2.496	.007 *	-.118	-2.048	.021 *
Household income	-	221	-.129	-1.915	.028 *	-.145	-2.156	.016 *	-.157	-2.343	.010 *
Mini-mental state examination	-	326	-.038	-0.686	.247	-.086	-1.544	.062	-.077	-1.389	.083
Geriatric depression scale	+	327	.032	0.571	.284	.031	0.553	.290	.036	0.646	.259
CES-Depression	+	327	-.037	-0.665	.747	-.001	-0.010	.504	-.013	-0.228	.590
Smoking status	+	278	.054	0.888	.188	.017	0.287	.387	.033	0.541	.294
Frequency of alcohol intake	+	163	-.049	-0.622	.732	-.076	-0.960	.831	-.083	-1.042	.851
Amount of alcohol intake	+	160	.071	0.880	.190	.034	0.424	.336	.049	0.614	.270
Frequency of 6 glasses of alcohol intake	+	161	.020	0.250	.401	.156	1.966	.026 *	.111	1.389	.083
Diabetes diagnosis	+	328	.013	0.231	.409	.075	1.353	.089	.059	1.070	.143
HOMA-Insulin resistance	+	318	.047	0.832	.203	.045	0.793	.214	.054	0.957	.170
Hemoglobin A1c	+	322	.020	0.349	.364	.031	0.545	.293	.034	0.609	.271
Fasting glucose	+	294	.040	0.686	.247	.134	2.303	.011 *	.113	1.933	.027 *
Post-load glucose	+	276	-.008	-0.138	.555	.084	1.387	.083	.059	0.972	.166
Body mass index	+	327	-.074	-1.327	.907	-.101	-1.816	.965	-.099	-1.793	.963
Diastolic blood pressure	+	281	.091	1.510	.066	.131	2.197	.014 *	.124	2.083	.019 *
Systolic blood pressure	+	281	.015	0.250	.401	.054	0.906	.183	.040	0.673	.251
Metabolic load factor	+	321	-.045	-0.808	.790	.039	0.702	.242	.010	0.170	.432
Gamma-glutamyltransferase	+	327	-.023	-0.405	.657	.049	0.889	.187	.022	0.401	.344
Uric acid	+	327	.047	0.835	.202	.086	1.554	.061	.079	1.423	.078
Tumor necrosis factor-alpha	+	307	.084	1.461	.073	.065	1.130	.130	.080	1.394	.082
**Cognition**											
Digit symbol substitution test	-	324	-.174	-3.150	9E-4 **	-.137	-2.467	.007 *	-.178	-3.230	7E-4 **
Episodic memory	-	335	-.095	-1.742	.041 *	-.136	-2.494	.007 *	-.138	-2.527	.006 *
Working memory	-	335	-.093	-1.702	.045 *	-.151	-2.783	.003 *	-.146	-2.678	.004 *
Fluid intelligence	-	335	-.075	-1.367	.086	-.136	-2.503	.006 *	-.129	-2.358	.009 *
**Life perspective**											
Future time perspective	-	332	.029	0.522	.699	.037	0.678	.751	.038	0.690	.755
Consideration of future consequences	-	335	.030	0.544	.706	-.032	-0.580	.281	-.011	-0.203	.420

Partial Pearson correlations were carried out with sex, age, and total intracranial volume serving as covariates. As we used three covariates, all t-values are specified by n-2-3 degrees of freedom.

h1: hypothesized effect direction (positive + or negative – association with brain age gap), grey matter: grey matter brain age gap, white matter: white matter brain age gap, grey & white matter: combined grey and white matter brain age gap, *n*: number of observations, *r*: Pearson's correlation coefficient, *t*: *t*-statistic, *p*: *p*–value (one-tailed)

* $p < .050$ (one-tailed nominal significance)
** $p < .001$ (one-tailed level of significance after multiple testing correction)

Associations of years of education and household income with brain age gap

Partial correlation analyses revealed nominally significant results between brain age gap and the criterion variables ‘years of education’ as well as ‘household income’ (Table A2). Moreover, the two criterion variables were observed to correlate with each other at $r = .29$ (Fig. A4). In order to examine if the two criterion variables independently correlate with brain age gap, we carried out an extended partial correlation analyses between each criterion variable and brain age gap by considering sex, age, age^2, total intracranial volume, and the respective other criterion variable as covariates (i.e., household income was used as covariate for associations of years of education and vice versa). Results are shown in Table A5. We observed that four out of five associations remained nominally significant when testing for independent associations, suggesting that the two criterion variables share unique variance with brain age gap. It should be noted that we followed the common approach of considering complete cases for partial correlations, so that sample sizes were smaller for the ‘extended’ partial correlations relative to the ‘default’ partial correlations (i.e., subjects with missing values in any of the considered variables *were excluded). Despite the lower statistical power for extended partial correlation analysis, the majority of observed associations remained nominally significant, providing support for independent associations between brain age gap and years of education as well as between brain age gap and household income.

Table A5 Unique associations of years of education and household income with brain age gap

			Grey matter			White matter			Grey and white matter		
	h1	*n*	*r*	*t*	*p*	*r*	*t*	*p*	*r*	*t*	*p*
**Partial correlations – default**[a]											
Years of education	-	300	-.033	-0.569	.285	-.143	-2.483	.007 *	-.116	-2.000	.023 *
Household income	-	221	-.131	-1.936	.027 *	-.145	-2.150	.016 *	-.158	-2.347	.010 *
**Partial correlations – extended**[b]											
Years of education	-	219	-.019	-0.276	.391	-.174	-2.567	.005 *	-.130	-1.914	.028 *
Household income	-	219	-.118	-1.727	.043 *	-.095	-1.393	.082	-.117	-1.710	.044 *

Partial Pearson correlations between brain age gap and criterion variables ‘years of education’ and ‘household income’. *t*-values are specified by n-2-4 (partial correlations – default) and n-2-5 (partial correlations – extended) degrees of freedom. *h1*: hypothesized effect direction (positive + or negative – association with brain age gap), grey matter: grey matter brain age gap, white matter: white matter brain age gap, grey and white matter: combined grey and white matter brain age gap, *n*: number of observations, *r*: Pearson’s correlation coefficient, *t*: *t*-statistic, *p*: *p*-value (one-tailed)

a correlations were adjusted by sex, age, age^2, and total intracranial volume. These results are identical to results presented in Table A3

b correlations were adjusted by sex, age, age^2, total intracranial volume, and one of the two criterion variables (i.e., household income was used as covariate for associations of years of education and vice versa).

$p < .050$ (one-tailed nominal significance)

Observed effect sizes vs. literature effect sizes

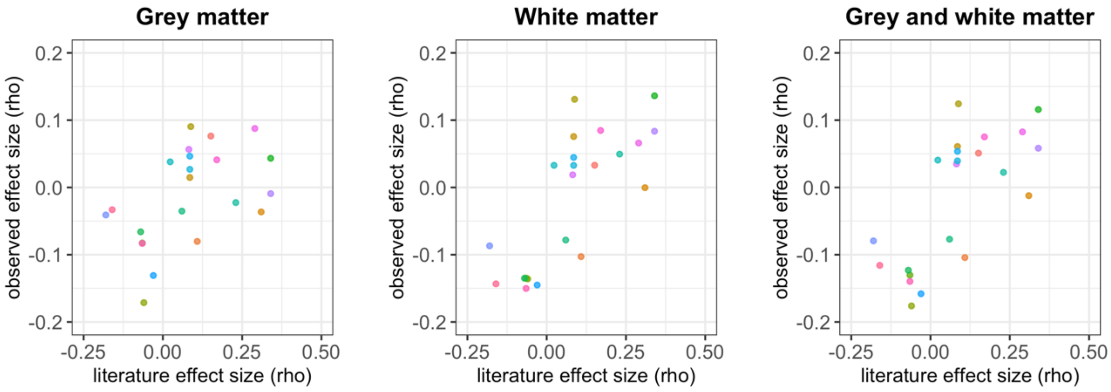

Fig. A8 Effect sizes of criterion variables observed in the present study (y-axis) plotted against respective effect sizes reported in the previous literature (x-axis; see suppl. Table B1 for details). If multiple literature effect sizes were available for a single criterion variable, the median effect size was calculated. The plots indicate a noticeable degree of consistency between the observed vs. literature effect sizes and directions, respectively. Overall, plots suggest a lower range of observed effect sizes when compared to effect sizes reported in the previous literature, i.e., effect sizes tended to be smaller in the present study.

References

Alberti, K. G. M. M., Eckel, R. H., Grundy, S. M., Zimmet, P. Z., Cleeman, J. I., Donato, K. A., et al. (2009). Harmonizing the metabolic syndrome: a joint interim statement of the International Diabetes Federation Task Force on Epidemiology and Prevention; National Heart, Lung, and Blood Institute; American Heart Association; World Heart Federation; International Atherosclerosis Society; and International Association for the Study of Obesity. Circulation 120, 1640–1645. doi:10.1161/CIRCULATIONAHA.109.192644.

Bertram, L., Böckenhoff, A., Demuth, I., Düzel, S., Eckardt, R., Li, S. C., et al. (2014). Cohort profile: The Berlin aging study II (BASE-II). Int. J. Epidemiol. 43, 703–712. doi:10.1093/ije/dyt018.

Champely, S. (2020). pwr: Basic Functions for Power Analysis. Available at: https://cran.r-project.org/package=pwr.

Düzel, S., Buchmann, N., Drewelies, J., Gerstorf, D., Lindenberger, U., Steinhagen-Thiessen, E., et al. (2018). Validation of a single factor representing the indicators of metabolic syndrome as a continuous measure of metabolic load and its association with health and cognitive function. PLoS One 13. doi:10.1371/journal.pone.0208231.

Düzel, S., Voelkle, M. C., Düzel, E., Gerstorf, D., Drewelies, J., Steinhagen-Thiessen, E., et al. (2016). The Subjective Health Horizon Questionnaire (SHH-Q): Assessing Future Time Perspectives for Facets of an Active Lifestyle. Gerontology 62, 345–353. doi:10.1159/000441493.

Folstein, M. F., Folstein, S. E., and McHugh, P. R. (1975). "Mini-mental state". A practical method for grading the cognitive state of patients for the clinician. J. Psychiatr. Res. 12, 189–198. doi:10.1016/0022-3956(75)90026-6.

Frick, J. R., Jenkings, S. P., Lillard, D. R., Lipps, O., and Wooden, M. (2007). The Cross-National Equivalent File (CNEF) and Its Member Country Household Panel Studies. Schmollers Jahrb. Zeitschrift für Wirtschafts- und Sozialwissenschaften 127, 627–654. Available at: http://hdl.handle.net/10419/67059.

Gayoso-Diz, P., Otero-González, A., Rodriguez-Alvarez, M. X., Gude, F., García, F., De Francisco, A., et al. (2013). Insulin resistance (HOMA-IR) cut-off values and the metabolic syndrome in a general adult population: effect of gender and age: EPIRCE cross-sectional study. BMC Endocr. Disord. 13, 47. doi:10.1186/1472-6823-13-47.

Goebel, J., Grabka, M. M., Liebig, S., Kroh, M., Richter, D., Schröder, C., et al. (2019). The German Socio-Economic Panel (SOEP). Jahrb. Natl. Okon. Stat. 239, 345–360. doi:doi:10.1515/jbnst-2018-0022.

Helmstaedter, C., and Durwen, H. F. (1990). VLMT: Verbaler Lern- und Merkfähigkeitstest: Ein praktikables und differenziertes Instrumentarium zur Prüfung der verbalen Gedächtnisleistungen. [VLMT: A useful tool to assess and differentiate verbal memory performance.]. Schweizer Arch. für Neurol. Neurochir. und Psychiatr. 141, 21–30.

Ho, D. E., Imai, K., King, G., and Stuart, E. A. (2011). {MatchIt}: Nonparametric Preprocessing for Parametric Causal Inference. J. Stat. Softw. 42, 1–28. Available at: https://www.jstatsoft.org/v42/i08/.

Idriss, H. T., and Naismith, J. H. (2000). TNF alpha and the TNF receptor superfamily: structure-function relationship(s). Microsc. Res. Tech. 50, 184–195. doi:10.1002/1097-0029(20000801)50:3<184::AID-JEMT2>3.0.CO;2-H.

Jaeger, J. (2018). Digit Symbol Substitution Test: The Case for Sensitivity Over Specificity in Neuropsychological Testing. J. Clin. Psychopharmacol. 38, 513–519. doi:10.1097/JCP.0000000000000941.

Kim, S. Y., Guevara, J. P., Kim, K. M., Choi, H. K., Heitjan, D. F., and Albert, D. A. (2010). Hyperuricemia and coronary heart disease: a systematic review and meta-analysis. Arthritis Care Res. (Hoboken). 62, 170–180. doi:10.1002/acr.20065.

Kooij, D. T. A. M., Kanfer, R., Betts, M., and Rudolph, C. W. (2018). Future time perspective: A

systematic review and meta-analysis. J. Appl. Psychol. 103, 867–893. doi:10.1037/apl0000306.

Lee, D. S., Evans, J. C., Robins, S. J., Wilson, P. W., Albano, I., Fox, C. S., et al. (2007). Gamma Glutamyl Transferase and Metabolic Syndrome, Cardiovascular Disease, and Mortality Risk. Arterioscler. Thromb. Vasc. Biol. 27, 127–133. doi:10.1161/01.ATV.0000251993.20372.40.

Lewinsohn, P. M., Seeley, J. R., Roberts, R. E., and Allen, N. B. (1997). Center for Epidemiologic Studies Depression Scale (CES-D) as a screening instrument for depression among community-residing older adults. Psychol. Aging 12, 277–287. doi:10.1037//0882-7974.12.2.277.

Lindenberger, U., Mayr, U., and Kliegl, R. (1993). Speed and intelligence in old age. Psychol. Aging 8, 207–220. doi:10.1037/0882-7974.8.2.207.

Mitchell, A. J. (2009). A meta-analysis of the accuracy of the mini-mental state examination in the detection of dementia and mild cognitive impairment. J. Psychiatr. Res. 43, 411–431. doi:10.1016/j.jpsychires.2008.04.014.

Mitchell, A. J., Bird, V., Rizzo, M., and Meader, N. (2010). Diagnostic validity and added value of the geriatric depression scale for depression in primary care: A meta-analysis of GDS30 and GDS15. J. Affect. Disord. 125, 10–17. doi:https://doi.org/10.1016/j.jad.2009.08.019.

Schacter, D. L., Osowiecki, D., Kaszniak, A. W., Kihlstrom, J. F., and Valdiserri, M. (1994). Source memory: Extending the boundaries of age-related deficits. Psychol. Aging 9, 81–89. doi:10.1037/0882-7974.9.1.81.

Schmiedek, F., Lövdén, M., and Lindenberger, U. (2010). Hundred Days of Cognitive Training Enhance Broad Cognitive Abilities in Adulthood: Findings from the COGITO Study. Front. Aging Neurosci. 2, 27. doi:10.3389/fnagi.2010.00027.

Strathman, A., Gleicher, F., Boninger, D. S., and Edwards, C. S. (1994). The Consideration of Future Consequences: Weighing Immediate and Distant Outcomes of Behavior. J. Pers. Soc. Psychol. 66, 742–752. doi:10.1037/0022-3514.66.4.742.

Wechsler, D., Corporation., P., and Pearson Education, I. (2008). WAIS-IV : Wechsler adult intelligence scale.

WHO (2004). Screening for Type 2 Diabetes: Report of a World Health Organization and International Diabetes Federation meeting. Diabetes Care 27, 5167–5170, 5175.

Yesavage, J. A., Brink, T. L., Rose, T. L., Lum, O., Huang, V., Adey, M., et al. (1982). Development and validation of a geriatric depression screening scale: A preliminary report. J. Psychiatr. Res. 17, 37–49. doi:10.1016/0022-3956(82)90033-4.
